# Identification of cardiovascular disease in patients with kidney stone disease using explainable machine learning

**DOI:** 10.3389/fcvm.2026.1696079

**Published:** 2026-05-29

**Authors:** Qinglong Yang, Nan Luo, Hanyuan Lin, Haolin Chen, Haoxian Tang, Jingtao Huang, Xuan Zhang, Wenqiang Liao, Yuxue Lin, Zexuan Liu, Xuxia Sui, Qingtao Yang, Gaoming Hou

**Affiliations:** 1 Department of Urology, The Second Affiliated Hospital of Shantou University Medical College, Shantou, Guangdong, China; 2 Shantou University Medical College, Shantou, Guangdong, China; 3 Department of Psychiatry, Shantou University Mental Health Center, Shantou, Guangdong, China; 4 Department of Thyroid, Breast and Hernia Surgery, General Surgery, The Second Affiliated Hospital of Shantou University Medical College, Shantou, Guangdong, China; 5 Department of Cardiology, The First Affiliated Hospital of Shantou University Medical College, Shantou, Guangdong, China; 6 Department of Sports Medicine and Rehabilitation, Peking University Shenzhen Hospital, Shenzhen, Guangdong, China; 7 Department of Bone & Joint Surgery, Peking University Shenzhen Hospital, Shenzhen, Guangdong, China; 8 Department of Pathogenic Biology, Shantou University Medical College, Shantou, Guangdong, China

**Keywords:** cardiovascular disease, kidney stones, machine learning, NHANES, SHAP

## Abstract

**Background:**

Kidney stone disease is an independent risk factor for cardiovascular disease (CVD), but specific tools for identifying CVD in patients with kidney stone disease are lacking. This study aimed to validate the association between kidney stones and CVD and to develop an interpretable machine learning model for the identification of prevalent CVD in individuals with kidney stones.

**Methods:**

Using data from 34,770 participants in the NHANES 2007–2018 cycle, weighted multivariable logistic regression and subgroup analysis were employed to examine the association between kidney stones and CVD. A total of 1,491 NHANES participants from 2007 to 2016 were used for model development and internal validation, while 296 participants from the 2017–2018 cycle were used as an independent temporal validation cohort. The Shapley Additive exPlanation (SHAP) method was used for global and local interpretation.

**Results:**

Model 3 revealed a 47% increased risk of CVD in participants with kidney stones compared to those without (OR = 1.47, 95% CI: 1.20–1.80). In the internal test set, the logistic regression (LR) model performed best, with an area under the receiver operating characteristic curve of 0.801, sensitivity of 0.721, specificity of 0.771, accuracy of 0.759, recall of 0.721, and Brier score of 0.169. LR also demonstrated the best performance in the temporal validation cohort. SHAP analysis identified the importance of 15 predictors.

**Conclusions:**

This study highlights an association between kidney stones and prevalent CVD, though causality cannot be inferred due to the cross-sectional design. The LR model demonstrated strong performance in identifying prevalent CVD in patients with kidney stone disease.

## 1 Introduction

Kidney stone disease is a common urinary system disorder, with epidemiological data showing a prevalence of 11.9% in men and 9.4% in women in the U.S. adult population in 2018 (1). The Global Burden of Disease study reported approximately 106 million new cases of kidney stones in 2021, reflecting a 26.7% increase since 2000 (2). Although surgical treatment effectively removes kidney stones, patients remain at high risk for recurrence (3). As the population ages, managing kidney stone patients has become a critical public health concern (2).

Cardiovascular disease (CVD), the leading cause of death and disability globally, accounted for about 18.6 million deaths in 2019 (4). Metabolic syndrome, a common risk factor for both CVD and kidney stones, plays a pivotal role. Characterized by hypertension, hyperglycemia, dyslipidemia, and central obesity, it significantly increases the risk of CVD (5) and is also strongly associated with kidney stone formation (6). These findings suggest that kidney stones are not only urological disorders but may also reflect systemic metabolic disturbances, sharing common pathophysiological mechanisms with CVD (7). Additionally, lifestyle and dietary factors commonly observed in kidney stone patients, including diet, physical inactivity, and smoking, are frequently linked to CVD (8). The association between kidney stones and CVD has gained increasing recognition. Studies have shown that kidney stones are a risk factor for CVD, including coronary heart disease, stroke, myocardial infarction, and congestive heart failure (7, 9–13). Consequently, risk assessment and early CVD intervention in kidney stone patients are clinically important.

Commonly used CVD risk assessment tools, such as the Framingham score (14) and pooled cohort equations risk score (15), while valuable in the general population, have three main limitations: first, they rely on invasive biomarkers (e.g., lipid profiles), restricting screening accessibility (16); second, they lack targeted models for specific populations, such as patients with kidney stones; and third, they fail to adequately incorporate modifiable factors (e.g., diet, physical activity) to guide preventive practices (16). Notably, dietary patterns and lifestyle choices are both key contributors to kidney stone formation and important modifiable risk factors for CVD (3, 4). Predictive models incorporating these variables could enhance assessment ease and provide targets for personalized behavioral interventions. In recent years, machine learning technology has shown distinct advantages in CVD risk prediction, successfully constructing high-precision models for specific populations, including those with type 2 diabetes (17), airflow obstruction (18), chronic lung disease (19), mental illness (20), and hyperlipidemia (21). Similarly, machine learning has gained increasing attention in nephrology, particularly for applications related to acute kidney injury and chronic kidney disease (CKD) (22, 23), where it shows significant potential as an innovative driver of progress in the field. Kidney stone disease, an independent CVD risk factor, affects a large patient population; however, no non-invasive model has been developed for identifying CVD in this population.

This study utilized data from the National Health and Nutrition Examination Survey (NHANES) 2007–2018 cycle to integrate non-invasive variables, such as demographic characteristics, lifestyle indicators, anthropometric parameters, and dietary nutrient intake. Machine learning algorithms were used to develop a model for identifying CVD in adult patients with kidney stone disease in the United States. Additionally, the study further validated the association between kidney stones and CVD through a large cohort analysis.

## 2 Methods

### 2.1 Data sources

The NHANES, administered by the National Center for Health Statistics (NCHS), is a nationally representative cross-sectional program aimed at assessing the health and nutritional status of U.S. residents who are not institutionalized. To ensure the collection of reliable and generalizable data, NHANES utilizes a multistage, stratified probability cluster sampling design. Each year, about 5,000 participants undergo structured interviews and standardized medical examinations, which gather a wide range of information, including demographic data, socioeconomic status, dietary habits, health history, physiological measurements, and laboratory test results (24).

### 2.2 Ethics

The NHANES protocol is approved by the NCHS Research Ethics Review Board, and written informed consent is obtained from all participants before their involvement in the study.

### 2.3 Study design and population

NHANES cycles from 2007 to 2018 were selected because key non-invasive variables, including dietary intake, lifestyle factors, and questionnaire-based measures, were collected with relatively consistent definitions and methodologies across these cycles. In contrast, the 2019–2020 cycle was affected by interruptions related to the COVID-19 pandemic, resulting in incomplete data and potential selection bias, while the 2021–2023 cycles currently lack kidney stone diagnosis data.

**Figure 1:**
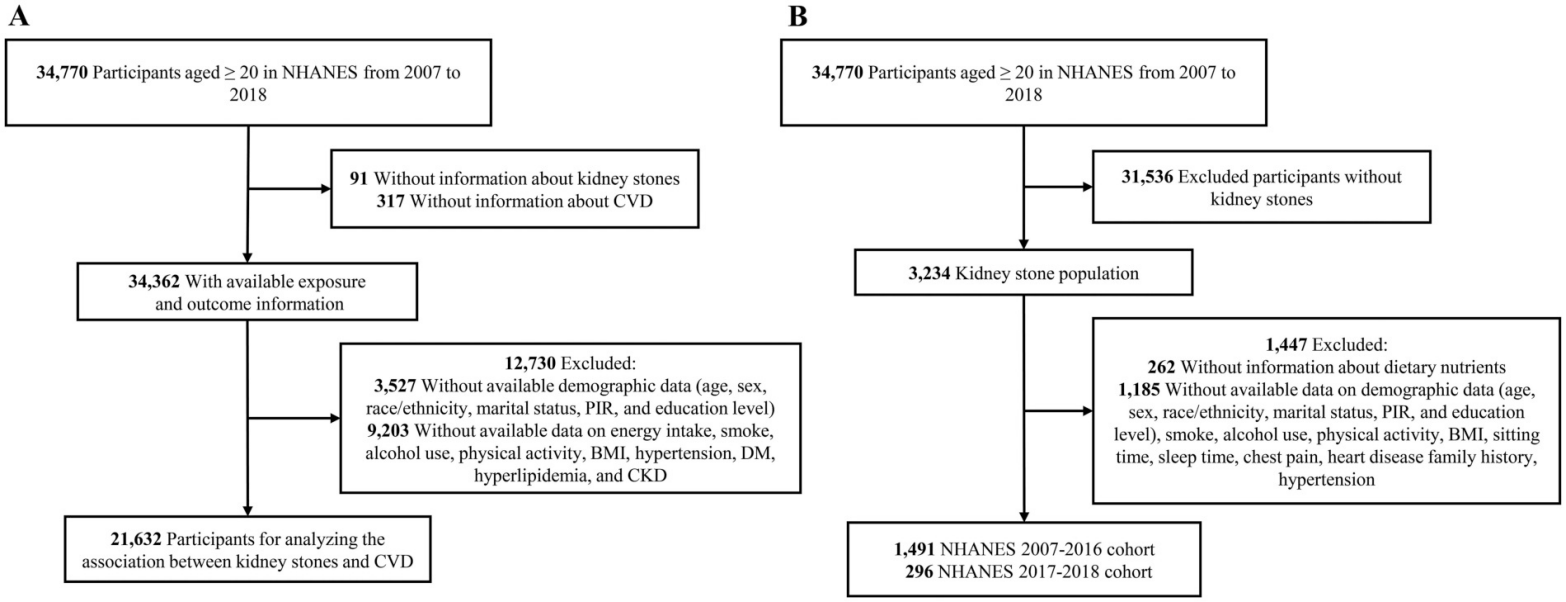
Flow diagram of the screening of study participants. **(A)** Flowchart of population selection for analyzing the association between kidney stones and CVD; **(B)** Flowchart of population selection for machine learning analysis of kidney stone participants. BMI, body mass index; CKD, chronic kidney disease; CVD, cardiovascular disease; DM, diabetes mellitus; PIR, poverty income ratio.

Our study comprised two populations. The first population included 34,770 NHANES 2007–2018 participants aged 20 years or older. We excluded 91 participants without information on kidney stones, 317 without data on CVD, and 12,730 lacking demographic data [age, sex, race/ethnicity, marital status, poverty income ratio (PIR), education level] and information on energy intake, smoking, alcohol use, physical activity, body mass index (BMI), hypertension, diabetes mellitus (DM), hyperlipidemia, and CKD. The final cohort for analysis of the association between kidney stones and CVD included 21,632 participants (Figure 1A).

The second population included 34,770 NHANES 2007–2018 participants aged 20 years or older. From this group, 31,536 participants without kidney stones were excluded, followed by 262 participants missing dietary nutrient data and 1,185 participants without demographic data, smoking, alcohol use, physical activity, BMI, sitting time, sleep time, chest pain, heart disease family history, or hypertension. No additional exclusion of extreme or outlier values in continuous variables was performed to preserve the natural distribution of the real-world NHANES dataset. The participants with kidney stones were then divided into two cohorts based on NHANES survey cycles: the 2007–2016 cohort (*N* = 1,491) was used for model development and internal validation, while the 2017–2018 cohort (*N* = 296) served as an independent temporal validation cohort (Figure 1B).

### 2.4 Diagnosis of CVD

CVD diagnosis was based on self-reported data (25). Participants were asked whether a doctor or other healthcare professional had ever informed them that they had any of the following conditions: congestive heart failure, coronary heart disease, angina (also referred to as angina pectoris), heart attack (also known as myocardial infarction), or stroke. If a participant answered affirmatively to any of these conditions, they were classified as having CVD (26).

### 2.5 Diagnosis of kidney stones

Kidney stones were diagnosed based on self-report (25). Participants were asked, “Have you ever had kidney stones?” Individuals who answered yes were classified as having kidney stones. The reliability of this diagnostic approach has been confirmed by previous studies (27).

### 2.6 Covariates and machine learning predictors

In the analysis of the total population, kidney stones served as the independent variable, and CVD was the dependent variable. Building on prior studies and clinical experience (28), 15 covariates were identified: age, sex, race/ethnicity, marital status, PIR, education level, energy intake, smoke, alcohol use, physical activity, BMI, hypertension, DM, hyperlipidemia, and CKD.

In developing a machine learning model for the identification of prevalent CVD in patients with kidney stone disease, we selected predictors based on previously identified covariates, excluding those that require invasive tests for diagnosis, such as DM, hyperlipidemia, and CKD. We also incorporated dietary nutrients, including macronutrients, minerals, and vitamins, as predictors. In total, 54 predictors were identified, covering a broad range of demographic variables, lifestyle factors, physical examination results, and dietary components. All these variables can be obtained without invasive tests, making the model highly actionable and convenient. For a detailed list of specific variables, please refer to Supplementary Table S1, and for definitions and grouping methods of covariates and predictors, see Supplementary Methodology 1.

### 2.7 Statistical analysis

In the total population analysis, mobile examination center (MEC) weights were applied. Participant characteristics were analyzed based on the presence or absence of CVD. Continuous variables were tested using the Wilcoxon rank-sum test for complex survey samples, and categorical variables using the Chi-squared test with Rao & Scott's second-order correction. Weighted multivariable logistic regression was used to examine the association between kidney stones and CVD. To mitigate potential bias, we employed 1:1 nearest-neighbor propensity score matching (PSM) to construct a comparable control group. Weighted multivariable logistic regression was then conducted on the matched data to assess the association between kidney stones and CVD. Three models were employed to adjust for covariates: Model 1 was unadjusted, Model 2 adjusted for age, sex, race/ethnicity, marital status, PIR, and education level, and Model 3 further adjusted for energy intake, smoking, alcohol use, physical activity, BMI, hypertension, DM, hyperlipidemia, and CKD. Subgroup analyses and interaction *P*-value calculations were performed to verify the stability of the results across different subgroups.

In the machine learning analysis, 54 predictors (demographic, lifestyle, and dietary nutrient variables) were initially considered. The machine learning analysis did not use weighted data because it did not need to consider the prevalence of the disease. For the specific reasons regarding the absence of weights in the machine learning analysis, refer to Supplementary Methodology 2. Intergroup differences were assessed using the Wilcoxon rank sum test for continuous variables and Pearson's Chi-squared test for categorical variables, based on the presence or absence of CVD.

The NHANES 2007–2016 cohort was used for model development and internal evaluation using nested resampling. Five-fold cross-validation was employed, with each fold sequentially serving as the internal test set in the outer loop, while the remaining folds underwent five-fold cross-validation in the inner loop for hyperparameter tuning. This nested approach ensures that every subset of the data has a chance to serve as a test set and provides a robust estimate of model performance. The NHANES 2017–2018 cohort was used as a temporal validation cohort to evaluate model performance. We employed both the least absolute shrinkage and selection operator (LASSO) regression and the Boruta algorithm for feature selection. LASSO uses an L1-norm penalty to shrink the coefficients of irrelevant features to zero, effectively identifying the most predictive variables. Boruta, a random forest–based algorithm, generates shadow features and compares their importance scores to the original features to determine their relevance. The combination of these complementary methods enabled us to robustly identify features most strongly associated with CVD in patients with kidney stone disease (29, 30). Feature selection was performed once on the full development cohort prior to nested cross-validation. For detailed information on the feature selection process, please refer to Supplementary Methodology 3. To address sample imbalance, the synthetic minority oversampling technique (SMOTE) was used to oversample the training set. For details on the implementation of SMOTE, refer to Supplementary Methodology 4.

To minimize bias in model evaluation, we employed 5-fold cross-validation to develop and assess five models in NHANES 2007–2016: logistic regression (LR), naive Bayes (NB), recursive partitioning and regression trees (RPART), k-nearest neighbors (KNN), and random forest (RF). We employed 5-fold cross-validation in the training set for hyperparameter tuning. Model performance was evaluated using metrics such as the receiver operating characteristic (ROC) curve's area under the curve (AUC), sensitivity, specificity, accuracy, recall, Brier score, F1 score, and balanced accuracy. The Brier score measures the discrepancy between predicted and actual outcomes, with lower values indicating better model performance. These metrics effectively reflect model performance (31). Additionally, ROC curves were plotted, and the model with the highest AUC was selected as the best model. We assessed the agreement between the optimal model's predicted and actual outcomes using a calibration curve and evaluated its clinical net benefit through decision curve analysis (DCA).

All models were externally validated using the NHANES 2017–2018 dataset. To evaluate class imbalance handling methods, optimal models employing over-sampling, under-sampling, and SMOTE were compared. The optimal model was further validated across demographic subgroups of kidney stone patients (sex, race, education, and marital status) to ensure broad applicability. Additionally, this model was applied to predict CVD risk in the general population and compared to its performance in the kidney stone cohort. To benchmark against a general CVD risk model, a LR model (Q-Lite) was developed using QRISK2-related predictors available in NHANES (32), including race, age, sex, smoke, systolic blood pressure, total cholesterol to high—density lipoprotein cholesterol ratio, PIR (substituting for the Townsend deprivation score), BMI, heart disease family history, hypertension, diabetes, CKD, and rheumatoid arthritis. Atrial fibrillation was excluded due to data unavailability. The performance of the Q-Lite model was compared with that of the study's optimal model, and their AUC values were statistically evaluated using the DeLong test. To assess the impact of sampling weights, we additionally built a weighted logistic regression model using NHANES survey weights. The model used the same features, data splits, and preprocessing steps as the unweighted model.

To enhance model interpretability, the Shapley Additive exPlanation (SHAP) method was employed to evaluate the decision-making patterns of the optimal model (selected through cross-validation). Grounded in Shapley values from cooperative game theory, SHAP quantifies the contribution of each feature to individual model outputs by calculating its average marginal impact across all possible feature combinations. This approach provides local and global explanations of feature influences on model outputs (33). We also utilized partial dependence plots to explore the relationship between individual features and model outputs.

In this study, data analysis was conducted using the statistical package R (version 4.4.1). The “survey” package (version 4.4–2) was employed for weighted analyses, the “mlr3” package (version 1.2.0) for machine learning model development and evaluation, and the “kernelshap” package (version 0.7.0) for interpretability analysis. A two-sided *P*-value < 0.05 was considered statistically significant.

## 3 Results

### 3.1 Characteristics of the participants

Table 1 presents the baseline characteristics of the total population of participants, stratified by the presence or absence of CVD. Of the 21,632 participants (weighted to represent 156,945,984 individuals), 19,221 had no CVD, and 2,411 had CVD. The weighted mean age (standard error) of participants was 48.24 (0.26) years, with 51.54% being female. Participants with CVD were more likely to be older, male, non-Hispanic White, have less than a high school education, engage in less physical activity, have a lower PIR, higher BMI, and higher rates of hypertension, DM, hyperlipidemia, CKD, and kidney stones compared to those without CVD. Supplementary Table S1 outlines the characteristics of participants with kidney stone disease, stratified by CVD status. Significant differences were observed between the two groups in all aspects, except for marital status, sleep time, cholesterol, vitamin A, retinol, folic acid, total choline, vitamin B12, vitamin C, vitamin D, iron, zinc, caffeine, and theobromine. After PSM, baseline characteristics were balanced between the CVD and non-CVD groups, each comprising 2,411 participants (Supplementary Table S2).

**Table 1:** Characteristics of participants in the NHANES 2007-2018 cycles.

Characteristic	Overall (*N* = 21,632)	Without CVD (*N* = 19,221)	With CVD(*N* = 2,411)	*P*-value
Weighted population	156,945,984	143,135,945	13,810,039	
Age, Mean (SE), years	48.24 (0.26)	46.63 (0.27)	64.93 (0.36)	<0.001
Age group, *n* (%)				<0.001
20–39	6,864 (33.94%)	6,768 (36.81%)	96 (4.22%)	
40–59	7,268 (38.41%)	6,752 (39.64%)	516 (25.61%)	
≥60	7,500 (27.65%)	5,701 (23.55%)	1,799 (70.17%)	
Sex, *n* (%)				<0.001
Female	11,073 (51.54%)	10,047 (52.21%)	1,026 (44.58%)	
Male	10,559 (48.46%)	9,174 (47.79%)	1,385 (55.42%)	
Race, *n* (%)				<0.001
Mexican American	3,029 (7.57%)	2,832 (7.95%)	197 (3.65%)	
Non-Hispanic Black	4,423 (10.03%)	3,900 (9.98%)	523 (10.62%)	
Non-Hispanic White	9,802 (70.47%)	8,435 (69.87%)	1,367 (76.62%)	
Other Hispanic	2,109 (5.14%)	1,923 (5.31%)	186 (3.39%)	
Other Race	2,269 (6.78%)	2,131 (6.89%)	138 (5.72%)	
Marital status, *n* (%)				<0.001
Married	11,338 (56.87%)	10,064 (56.82%)	1,274 (57.47%)	
Never married	3,811 (17.22%)	3,640 (18.24%)	171 (6.57%)	
Living with partner	1,703 (7.69%)	1,599 (7.96%)	104 (4.91%)	
Other	4,780 (18.22%)	3,918 (16.98%)	862 (31.05%)	
PIR, Mean (SE)	3.07 (0.04)	3.11 (0.04)	2.68 (0.05)	<0.001
Education level, *n* (%)				<0.001
Less than high school	4,638 (13.63%)	3,929 (12.95%)	709 (20.67%)	
High school or equivalent	4,963 (22.91%)	4,320 (22.36%)	643 (28.65%)	
Above high school	12,031 (63.46%)	10,972 (64.69%)	1,059 (50.68%)	
Energy intake, mean (SE), kcal/d	2,101.94 (8.71)	2,119.09 (9.06)	1,924.20 (20.40)	<0.001
smoke, *n* (%)				<0.001
Never	11,960 (55.74%)	11,029 (57.45%)	931 (38.03%)	
Former	5,456 (25.83%)	4,488 (24.37%)	968 (40.94%)	
Now	4,216 (18.43%)	3,704 (18.18%)	512 (21.03%)	
Alcohol use, *n* (%)				<0.001
Never	2,829 (10.01%)	2,527 (10.01%)	302 (10.00%)	
Former	3,999 (15.34%)	3,182 (13.84%)	817 (30.93%)	
Now	14,804 (74.65%)	13,512 (76.16%)	1,292 (59.07%)	
Physical activity, Mean (SE), min/week	937.81 (18.70)	972.37 (19.76)	579.60 (35.72)	<0.001
BMI, Mean (SE), kg/m^2^	29.23 (0.09)	29.06 (0.09)	30.97 (0.21)	<0.001
Hypertension, *n* (%)				<0.001
No	12,190 (61.49%)	11,698 (65.08%)	492 (24.29%)	
Yes	9,442 (38.51%)	7,523 (34.92%)	1,919 (75.71%)	
DM, *n* (%)				<0.001
No	17,448 (85.44%)	16,083 (87.79%)	1,365 (61.15%)	
Yes	4,184 (14.56%)	3,138 (12.21%)	1,046 (38.85%)	
Hyperlipidemia, *n* (%)				<0.001
No	5,981 (28.65%)	5,721 (30.57%)	260 (8.78%)	
Yes	15,651 (71.35%)	13,500 (69.43%)	2,151 (91.22%)	
CKD, *n* (%)				<0.001
No	17,643 (85.27%)	16,331 (87.61%)	1,312 (61.02%)	
Yes	3,989 (14.73%)	2,890 (12.39%)	1,099 (38.98%)	
Kidney stones, *n* (%)				<0.001
No	19,471 (89.66%)	17,491 (90.47%)	1,980 (81.20%)	
Yes	2,161 (10.34%)	1,730 (9.53%)	431 (18.80%)	

BMI, body mass index; CKD, chronic kidney disease; CVD, cardiovascular disease; DM, diabetes mellitus; NHANES, National Health and Nutrition Examination Survey; PIR, poverty income ratio; SE, standard error. Continuous variables were reported as mean and SE, whereas categorical variables were presented as number and frequency (%). All means and SEs for continuous variables and percentages for categorical variables were weighted.

### 3.2 Association between kidney stones and CVD

Table 2 illustrates the association between kidney stones and CVD. In Model 3, participants with kidney stones had a 47% increased risk of developing CVD compared to those without kidney stones (OR = 1.47, 95% CI: 1.20–1.80). The significant association between kidney stones and increased CVD risk persisted after matching (Table 2). Subgroup analyses revealed a significant interaction between kidney stones and CVD in the DM subgroup (*P* for interaction = 0.04; Supplementary Table S3). Specifically, the association was significant in the DM population (OR = 1.85, 95% CI: 1.40–2.45), while no significant association was found in the population without DM (OR = 1.25, 95% CI: 0.98–1.61).

**Table 2:** Multivariable logistic regression of association between kidney stones and CVD.

PSM status	Variable	Model 1^a^		Model 2^b^		Model 3^c^	
		OR(95%CI)	*P*-value	OR(95%CI)	*P*-value	OR(95%CI)	*P*-value
Before PSM	Kidney stones						
	No	1[Reference]		1[Reference]		1[Reference]	
	Yes	2.20 (1.85, 2.61)	<0.001	1.66 (1.35, 2.05)	<0.001	1.47 (1.20, 1.80)	<0.001
PSM	Kidney stones						
	No	1[Reference]		1[Reference]		1[Reference]	
	Yes	1.43 (1.22, 1.68)	<0.001	1.41 (1.21, 1.66)	<0.001	1.41 (1.21, 1.66)	<0.001

BMI, body mass index; CKD, chronic kidney disease; CI, Confidence Interval; CVD, cardiovascular disease; DM, diabetes mellitus; OR, Odd Ratio; PIR, poverty income ratio; PSM, Propensity Score Matching.

a Crude model.

b Adjusted for age, sex, race/ethnicity, marital status, PIR, education level.

c Adjusted for age, sex, race/ethnicity, marital status, PIR, education level, energy intake, smoke, alcohol use, physical activity, BMI, hypertension, DM, hyperlipidemia, and CKD.

### 3.3 Development and comparison of models for identifying CVD

**Figure 2:**
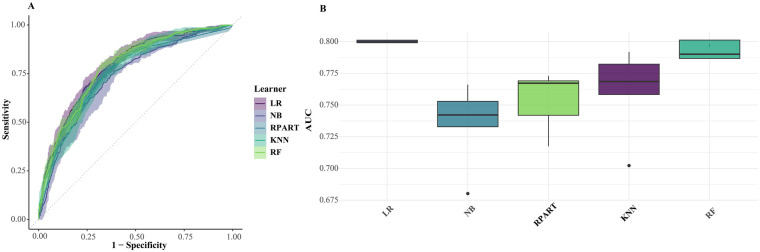
Receiver operating characteristic curves for the five machine learning models in identifying prevalent CVD in patients with kidney stone disease. **(A)** Receiver operating characteristic curves; **(B)** Areas under the receiver operating characteristic curves. AUC, Area Under the Curve; KNN, k-nearest neighbors; LR, logistic regression; NB, naive Bayes; RF, random forest; RPART, recursive partitioning and regression trees.

Initially, 54 predictors were considered for machine learning model development. The Boruta algorithm identified 30 significant and 9 tentatively important variables (Supplementary Figure S1), while LASSO regression selected 18 potential predictors (Supplementary Figure S2). Based on the overlap between the two methods and clinical relevance, 15 features were ultimately selected for model development: age, sex, race, PIR, education level, physical activity, BMI, smoke, sitting time, hypertension, chest pain, family history of heart disease, magnesium, polyunsaturated fatty acids (PUFA), and folate. Five machine learning models were constructed to identify CVD risk in participants with kidney stones. Figure 2 displays the internal test set ROC curves for the five models, and Table 3 presents the average values of the detailed performance metrics of these models for the internal test set. The LR model performed well with an AUC of 0.801, sensitivity of 0.721, specificity of 0.771, accuracy of 0.759, recall of 0.721, Brier score of 0.169, F1 score of 0.590, and Balanced accuracy of 0.746. The RF model also showed strong performance with an AUC of 0.790, sensitivity of 0.651, specificity of 0.768, accuracy of 0.739, recall of 0.651, Brier score of 0.169, F1 score of 0.546, and Balanced accuracy of 0.709. Model performance was ranked by AUC as follows: LR, RF, KNN, RPART, and NB. Ultimately, the LR model was selected to identify prevalent CVD in patients with kidney stone disease. Supplementary Table S4 lists the average values of the detailed performance metrics of the model on the training set. The calibration curve indicated that the predicted probabilities of the LR model closely aligned with the observed outcomes (Supplementary Figure S3). DCA demonstrated that the LR model provided greater net benefits than the Q-Lite model (Supplementary Figure S4). Table 4 presents model performance metrics in the temporal validation cohort, where the LR model achieved the highest AUC of 0.803. Supplementary Figure S5 presents the confusion matrix of the LR model applied to the temporal validation cohort. Supplementary Table S5 compares class imbalance methods, showing that LR with SMOTE (AUC = 0.801) outperformed over-sampling (AUC = 0.766) and under-sampling (AUC = 0.784). Supplementary Table S6 presents the performance of the logistic regression model across different demographic subgroups. Overall, balanced accuracy remained relatively consistent across subgroups, whereas other performance metrics showed variability. In sex-stratified analyses, the model demonstrated higher sensitivity in males (0.727 vs. 0.585) and higher specificity in females (0.842 vs. 0.704), with similar balanced accuracy between the two groups (0.716 vs. 0.714). Across racial subgroups, the model achieved higher AUC values in non-Hispanic Black (0.835) and Mexican American participants (0.826), compared with other Hispanic groups (0.732). In education-level stratification, performance also varied, with lower education groups showing higher sensitivity but lower specificity compared with higher education groups. Supplementary Table S7 reports predictive metrics of the optimal LR model in the general population, with an AUC of 0.765, lower than the 0.801 observed in the kidney stone cohort. Supplementary Table S8 shows that the Q-Lite model achieved an AUC of 0.781, slightly below that of the LR model in this study. The DeLong test indicated that the difference between their AUC values was not statistically significant (Supplementary Table S8). Supplementary Table S9 shows that the weighted LR model had slightly lower overall performance than the unweighted model (AUC: 0.758 vs. 0.801; F1 score: 0.543 vs. 0.590; balanced accuracy: 0.724 vs. 0.746). It also showed higher specificity (0.844 vs. 0.771) but lower sensitivity (0.604 vs. 0.721), indicating improved identification of negative cases at the expense of reduced detection of positive cases.

**Table 3:** Internal test set metrics for five machine learning models identifying prevalent CVD in patients with kidney stone disease.

Model	AUC	Sensitivity	Specificity	Accuracy	Recall	Brier Score	F1 score	Balanced accuracy
LR	0.801	0.721	0.771	0.759	0.721	0.169	0.590	0.746
NB	0.735	0.740	0.642	0.665	0.740	0.239	0.517	0.691
RPART	0.754	0.681	0.703	0.698	0.681	0.196	0.519	0.692
KNN	0.761	0.772	0.604	0.645	0.772	0.219	0.511	0.688
RF	0.790	0.651	0.768	0.739	0.651	0.169	0.546	0.709

AUC, area under the curve; KNN, k-nearest neighbors; LR, logistic regression; NB, naive bayes; RF, random forest; RPART, recursive partitioning and regression trees.

**Table 4:** Performance metrics of five machine learning models for identifying prevalent CVD in patients with kidney stone disease in the NHANES 2017–2018 temporal validation cohort.

Model	AUC	Sensitivity	Specificity	Accuracy	Recall	Brier Score	F1 score	Balanced accuracy
LR	0.803	0.742	0.756	0.753	0.742	0.173	0.573	0.749
NB	0.582	0.623	0.574	0.586	0.623	0.298	0.406	0.599
RPART	0.744	0.681	0.703	0.698	0.681	0.196	0.519	0.692
KNN	0.783	0.536	0.872	0.796	0.536	0.154	0.544	0.704
RF	0.791	0.695	0.630	0.645	0.696	0.217	0.471	0.663

AUC, area under the curve; KNN, k-nearest neighbors; LR, logistic regression; NB, naive bayes; RF, random forest; RPART, recursive partitioning and regression trees.

### 3.4 Model interpretation

**Figure 3:**
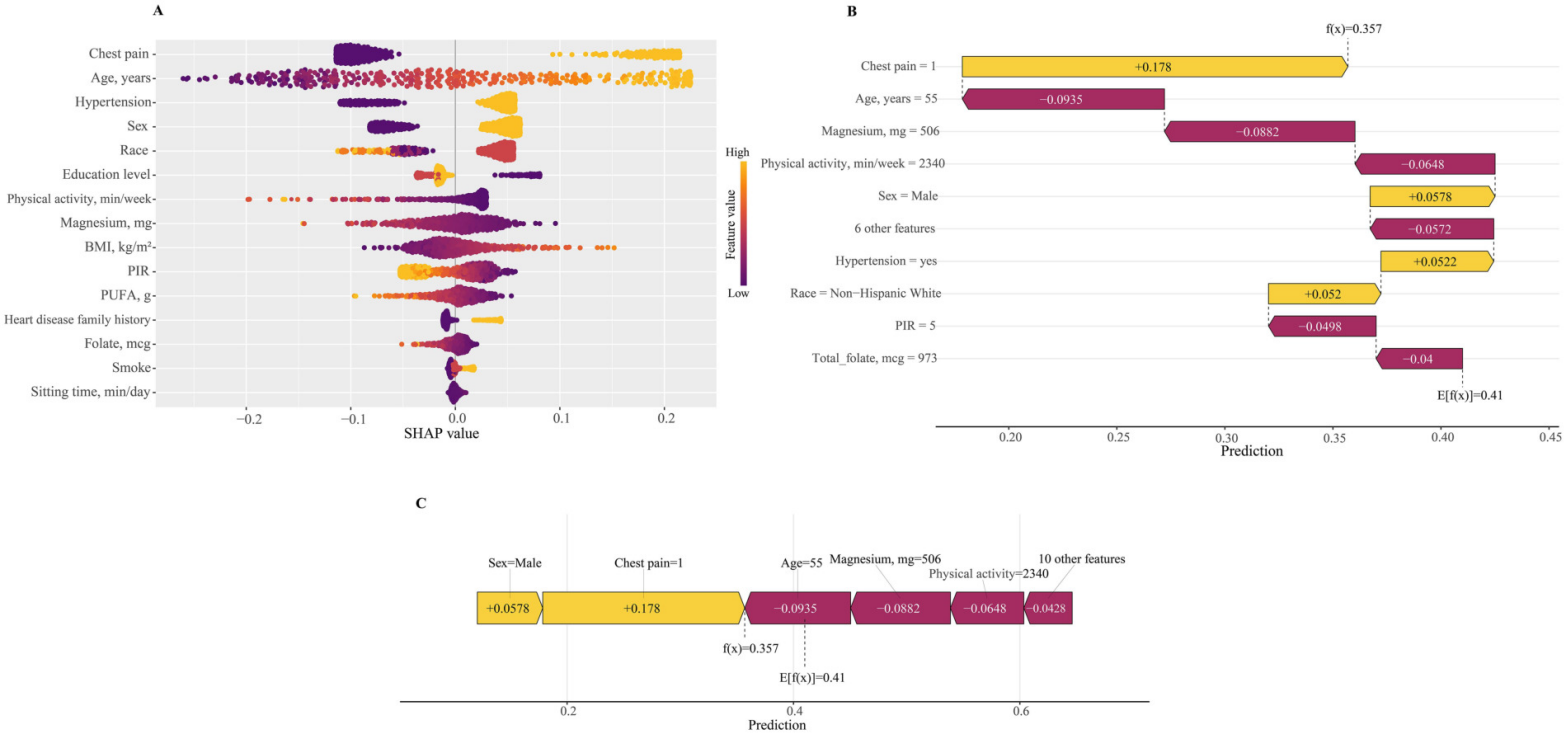
SHAP-based explanation of machine learning models. **(A)** SHAP summary dot plot; **(B)** SHAP waterfall plot; **(C)** SHAP force plot. BMI, body mass index; PIR, poverty income ratio; SHAP, Shapley Additive exPlanations; PUFA, polyunsaturated fatty acids.

We used the SHAP method to interpret the output of the LR model, providing both global and local interpretations. In the global interpretation, the SHAP summary dot plot (Figure 3A) illustrates the direction and strength of each feature's influence on the model's output for identifying CVD. The 15 predictors were ranked based on their contribution to the predicted outcome as follows: chest pain, age, hypertension, sex, race, education level, physical activity, magnesium, BMI, PIR, PUFA, heart disease family history, folate, smoke, and sitting time. Chest pain, age, hypertension, and BMI were the main positive influences, while education level, physical activity, magnesium, PIR, PUFA, and folate had negative influences.

In the local interpretation, the SHAP waterfall plot (Figure 3B) and SHAP force plot (Figure 3C) show the contribution of features for an individual without CVD. Yellow indicators represent positive influences on the model output, while red indicators represent negative influences. For instance, chest pain contributed positively (+0.178), while being aged 55 contributed negatively (−0.0935) to the model output. Based on the contributions of all features, the LR model's output for this patient leans towards being CVD-free, consistent with the actual outcome. Partial dependence plots revealed that age, BMI, and sitting time were positively associated with SHAP values, indicating a higher predicted risk with increasing values (Supplementary Figure S6). In contrast, physical activity, PIR, PUFA, folate, and magnesium showed negative associations with SHAP values. Features like hypertension, chest pain, heart disease family history, smoking, and male gender have higher SHAP values (Supplementary Figure S6).

## 4 Discussion

Our study found that kidney stones significantly increase the risk of CVD, with subgroup analyses revealing a stronger association in individuals with comorbid DM. AUC was selected as the primary evaluation metric due to its superior ability to assess model discrimination. For a comprehensive explanation of this choice and the comparative significance of other evaluation metrics, please refer to Supplementary Discussion 1. The LR model, which achieved the highest AUC in both internal validation and the temporal validation cohort, was identified as the optimal model. Feature importance analysis using SHAP values identified the 15 predictors of CVD risk, in order: chest pain, age, hypertension, sex, race, education level, physical activity, magnesium, BMI, PIR, PUFA, heart disease family history, folate, smoke, and sitting time. We assessed the stability of the LR model across demographic subgroups, confirming its broad applicability. The LR model demonstrated significantly better performance in the identification of prevalent CVD in patients with kidney stone disease compared to the general population. The general CVD risk assessment model (Q-Lite) showed lower performance in the kidney stone cohort than the LR model specifically developed for this group. We additionally performed a weighted analysis, which showed no improvement in performance over the unweighted model, supporting the use of the unweighted model as the primary approach.

Over the past decade, the relationship between kidney stones and CVD risk has become a key area of medical research. In 2013, Ferraro et al. (7) analyzed three prospective studies in the United States and found that a history of kidney stones significantly increased the risk of coronary heart disease in women (HR = 1.48). In 2014, Liu et al. (9) confirmed the association between kidney stones and an elevated risk of coronary heart disease (HR = 1.19) and stroke (HR = 1.40) through a meta-analysis of six cohort studies, suggesting that kidney stones may represent a systemic pathological condition affecting cardiovascular health. A long-term follow-up study by Alexander et al. (10), involving over 3 million people in Canada, found a similar link between kidney stones and increased risks of myocardial infarction (HR = 1.40) and stroke (HR = 1.26). A Mendelian randomization study conducted in 2021 provided genetic evidence linking kidney stones to an increased risk of CVD (34). This research suggests that genetic susceptibility to kidney stones may elevate the risk of coronary atherosclerosis and cardiomyopathy, thereby reinforcing the argument for a causal relationship. A recent cross-sectional study in Iran also found that patients with kidney stones had a 22% higher risk of CVD (including coronary heart disease, myocardial infarction, and stroke) compared to those without kidney stones (11). In this study, we validated the positive association between kidney stones and CVD risk using the NHANES database, with the additional finding that the association was more pronounced in DM patients. Given that DM is an independent risk factor for CVD, it may synergize with kidney stones through shared pathophysiological mechanisms, such as metabolic disturbances and inflammation, thus amplifying CVD risk (17). Current research suggests that kidney stones contribute to CVD through mechanisms like vascular calcification, oxidative stress mediated by osteoblast protein, inflammatory responses, and endothelial dysfunction (12). However, these mechanisms remain incompletely understood, and further research is needed to clarify the underlying processes.

Several studies have developed predictive models for CVD risk with good performance, including those by Hippisley-Cox et al. (35), D'Agostino Sr et al. (36), Yang et al. (37), Asadi et al. (38), Dorraki et al. (39), and Shen et al. (40). However, most existing models rely on laboratory test data, which not only increases healthcare costs but may also limit practical application due to patient reluctance to undergo testing. In contrast, models based on non-laboratory data, such as dietary nutrients, offer substantial advantages in resource-limited settings, reducing healthcare costs and promoting self-health management (16, 41). Moreover, studies have highlighted limitations in the generalizability of CVD risk assessment models for specific populations. For example, Dziopa et al. noted that traditional CVD models are poorly predictive for diabetic patients, likely due to the unique pathophysiological characteristics of diabetes not being adequately considered in these models (17). Other studies have addressed similar issues by developing specialized models for psychiatric populations (20), hyperlipidemia patients (21), populations with airflow obstruction (18), and chronic lung disease (19). These findings emphasize the need for tailored CVD risk assessment models for specific populations to improve classification performance and enable more precise prevention and intervention strategies. In contrast, this study focuses on patients with kidney stone disease, developing a model for identifying prevalent CVD using only non-laboratory examination data and demonstrating strong performance. This approach offers a feasible solution for CVD risk assessment in resource-constrained settings and provides a useful reference for expanding the applicability of predictive models and optimizing their architecture in the future. For a detailed explanation of why patients with kidney stone disease require an independent model for prevalent CVD identification, please see Supplementary Discussion 2. We suggest that future CVD identification approaches should incorporate multidimensional factors such as diet and lifestyle to better capture the diverse information influencing CVD burden, thereby improving model performance and practical utility. In addition to overall model performance, we further evaluated its consistency across population subgroups. Subgroup analyses revealed that model performance was not entirely uniform across demographic groups. Although balanced accuracy was relatively consistent, other metrics showed variability. For example, the model demonstrated higher sensitivity in males but higher specificity in females, indicating differences in the balance between identifying positive and negative cases across sex groups. Similarly, performance varied across racial and educational subgroups, with higher AUC observed in non-Hispanic Black and Mexican American participants compared with other groups. These findings suggest that the model may exhibit differential behavior across population subgroups, potentially reflecting differences in disease presentation, healthcare access, or reporting patterns. Therefore, while the model shows overall robustness, its performance in specific subpopulations should be interpreted with caution, and further validation in diverse cohorts is warranted.

As far as we are aware, our study is the first to develop a machine learning model for the identification of prevalent CVD, specifically in individuals with kidney stone disease. SHAP values quantify the contribution of each feature to model outputs, while partial dependence plots visualize the relationship between feature values and model outputs, aiding in model interpretation. Our analysis identified chest pain, age, and hypertension as major contributors to the identification of prevalent CVD. Chest pain is a commonly reported symptom in individuals with CVD, often associated with myocardial ischemia or cardiovascular conditions (42). Its high SHAP value reflects its contribution to the model-based identification of prevalent CVD. This may be particularly relevant in patients with kidney stone disease, who frequently experience pain symptoms, making chest pain more likely to be documented. Our findings suggest that chest pain may serve as a clinical indicator for patients with kidney stone disease and previously unrecognized CVD, assisting in case identification rather than implying future cardiovascular risk prediction. Age is a non-modifiable factor associated with CVD status. With aging, vascular elasticity decreases, vessel walls thicken, and cardiac function declines, increasing the likelihood of conditions such as coronary artery disease, heart failure, and atherosclerosis (43). Our results demonstrate a positive association between age and prevalent CVD, consistent with epidemiological evidence (44). In patients with kidney stones, age-related risk may be further complicated by metabolic syndrome, chronic kidney disease, and medication use, reinforcing age's predictive relevance (6). Hypertension is a major independent risk factor for CVD. Persistent high blood pressure impairs endothelial function and accelerates atherosclerosis, substantially increasing the risk of cardiovascular events (45). The high SHAP values associated with hypertension indicate its strong contribution to the identification of prevalent CVD, in line with findings from previous cohort studies (46). In kidney stone patients, the high prevalence of hypertension may stem from renal dysfunction and metabolic disturbances (47), which impair blood pressure regulation and amplify its impact on CVD risk.

For BMI and sitting time, SHAP values increased with higher feature values, indicating a positive association with model outputs for prevalent CVD. Elevated BMI reflects obesity, which is associated with CVD. Prolonged sedentary behavior may contribute to metabolic disorders and vascular dysfunction, which are associated with CVD (4). Sedentary behavior is prevalent among patients with kidney stone disease and is associated with metabolic disturbances such as insulin resistance, hyperglycemia, and dyslipidemia, which may contribute to CVD (48, 49). Additionally, sedentary behavior can reduce shear rates, potentially leading to endothelial dysfunction (50), and is linked to elevated inflammation and reactive oxygen species, further impairing vascular health (51). In contrast, SHAP values for physical activity, PIR, PUFA, folate, and magnesium were inversely related to feature values. Regular physical activity promotes cardiovascular health and lowers disease risk (4). PIR reflects socioeconomic status; lower PIR is often associated with limited healthcare access and unhealthy behaviors, increasing CVD risk (18). Our analysis indicates that insufficient intake of magnesium, PUFA, and folate is associated with higher model outputs for prevalent CVD, consistent with prior research (52–54). Magnesium, primarily sourced from green vegetables, grains, and legumes, may protect cardiovascular health through several mechanisms, including reducing chronic inflammation, enhancing antioxidant enzyme activity to neutralize free radicals, and promoting nitric oxide release to improve endothelial function (52). PUFA, found in foods such as vegetable oils, nuts, and oily fish, may be associated with lower CVD burden by influencing inflammation and lipid metabolism (53, 55). Folate, primarily obtained from fruits and vegetables or supplements, protects the cardiovascular system by reducing blood homocysteine levels, which helps lower hyperhomocysteinemia, an independent risk factor for CVD, thereby reducing CVD risk (54). Additionally, socio-demographic factors such as sex, race, education level, PIR, and family history of heart disease showed significant associations with prevalent CVD, consistent with findings from other studies (18). These factors likely influence CVD development by affecting lifestyle choices, healthcare access, and chronic disease management. Supplementary Table S10 summarizes the clinical significance and underlying mechanisms of the predictor variables.

This study provides multidimensional insights into factors associated with prevalent CVD in patients with kidney stone disease through interpretable machine learning models. We identified modifiable factors—hypertension, physical activity, magnesium, BMI, PUFA, folate, smoke, and sitting time—and non-modifiable factors—chest pain, age, sex, race, education level, PIR, and heart disease family history. Based on these factors, we propose targeted recommendations to prevent CVD in populations at risk for kidney stones. In terms of diet, we recommend increasing the intake of magnesium, PUFA, and folate through foods such as vegetables, fruits, legumes, and oily fish. It is also crucial to maintain a healthy lifestyle by increasing physical activity, reducing sedentary behavior, and quitting smoking. Regular medical checkups and monitoring of key health indicators, such as blood pressure and BMI, are essential, particularly for those at high risk of CVD, including individuals with chest pain, the elderly, and male patients. Notably, these prevention strategies also positively affect kidney stone prevention, reflecting the synergistic impact of dietary and lifestyle interventions in managing multiple chronic diseases (3). This approach enhances public health awareness and self-management, and promotes overall improvements in health literacy.

However, our study has several limitations. The diagnoses of kidney stones and CVD were based on self-reported data from interview questionnaires, which may be subject to recall bias. We validated the accuracy of self-reported CVD using ICD-10 diagnosis codes from NHANES prescription drug data. Among the 56 participants diagnosed with CVD, 53 (95%) self-reported the condition, while 3 did not. Details of the validation method and potential self-reporting biases are provided in Supplementary Discussion 3. Due to the cross-sectional design of NHANES, the temporal sequence between kidney stones and CVD onset cannot be clearly established, limiting causal inference. Although statistical methods such as PSM help reduce confounding, unmeasured confounders, and the lack of temporal data continue to constrain causal interpretation. Therefore, future longitudinal cohort studies are warranted. While a temporal validation cohort was used to validate model performance, the study was limited to the U.S. population. Given regional differences in diet and lifestyle, further validation in diverse populations is necessary to assess generalizability.

## 5 Conclusions

This study highlights an association between kidney stones and prevalent CVD based on U.S. adult data. By comparing five machine learning algorithms, we identified the LR model as the best for identifying prevalent CVD in patients with kidney stone disease. SHAP analysis highlighted the importance of diet and lifestyle factors in relation to CVD status, providing a foundation for targeted prevention strategies in patients with kidney stone disease.

## Data Availability

Publicly available datasets were analyzed in this study. This data can be found here: https://www.cdc.gov/nchs/nhanes.

## Supplementary material

The Supplementary Materialfor this article can be found online at: https://www.frontiersin.org/articles/10.3389/fcvm.2026.1696079/full#supplementary-material
