# Supplementary material for: Identification of cardiovascular disease in patients with kidney stone disease using explainable machine learning
Source: Front Cardiovasc Med. 2026 May 29;13:1696079. doi: 10.3389/fcvm.2026.1696079 (PMC13260074; doi:10.3389/fcvm.2026.1696079)
Supplement: Supplementary file 1 [file Supplementaryfile1.docx]

**Supplementary Materials Files:**

**Supplementary Text**

Supplementary Methodology 1. Definition and grouping of study variables.

Supplementary Methodology 2. Rationale for not using NHANES sampling weights in machine learning modeling.

Supplementary Methodology 3. Feature Selection Strategy and Implementation.

Supplementary Methodology 4. Handling class imbalance with SMOTE.

Supplementary Discussion 1. Explanation for selecting AUC as the primary metric and the clinical implications of various indicators.

Supplementary Discussion 2. Justification for developing a dedicated model for identifying prevalent CVD in patients with kidney stone disease.

Supplementary Discussion 3. Validation of self-reported CVD and potential self-reporting bias.

**Supplementary Figure**

Supplementary Figure 1. Feature selection with Boruta.

Supplementary Figure 2. Lasso regression-based variable screening.

Supplementary Figure 3. Calibration curve of LR model on internal test set.

Supplementary Figure 4. Decision curve analysis for LR Model and Q-Lite model on internal test set.

Supplementary Figure 5. Confusion matrix plot for LR model in the temporal validation cohort.

Supplementary Figure 6. Partial dependence plots.

**Supplementary Table**

Supplementary Table 1. Characteristics of patients with kidney stone disease in NHANES 2007 - 2018 cycles.

Supplementary Table 2. Characteristics of participants in the NHANES 2007-2018 cycles after propensity score matching.

Supplementary Table 3. Association between kidney stones and CVD in different subgroups.

Supplementary Table 4. Training set metrics for five machine learning models identifying prevalent CVD in patients with kidney stone disease.

Supplementary Table 5. Performance of logistic regression models for identifying prevalent CVD in patients with kidney stone disease using over-sampling and under-sampling preprocessing.

Supplementary Table 6. Performance metrics of the logistic regression model for identifying prevalent CVD across different demographic groups.

Supplementary Table 7. Performance metrics of the logistic regression model for identifying prevalent CVD in the general population.

Supplementary Table 8. Performance metrics of the Q-Lite model for identifying prevalent CVD in patients with kidney stone disease

Supplementary Table 9. Performance comparison of weighted and unweighted logistic regression models for identifying prevalent CVD in patients with kidney stone disease.

Supplementary Table 10. Clinical significance and interpretation of predictive variables.

**Supplementary Methodology 1. Definition and grouping of study variables**

Age was grouped into three categories: younger than 40, 40-59 years, and 60 years or older. Participants self-reported their race/ethnicity, with categories including Mexican American, non-Hispanic black, non-Hispanic white, other Hispanic, and other. Marital status was classified into four groups: married, never married, living with a partner, and other, which encompassed widowed, divorced, or separated individuals. The poverty-income ratio (PIR) was determined by dividing family or individual income by the poverty threshold. Education level was categorized into three groups: less than high school, high school or equivalent, and above high school. Body mass index (BMI) was calculated by dividing weight in kilograms by height in meters squared. Physical activity was measured as the total minutes of exercise per week, which included four activity types: walking or bicycling, tasks around the home or yard, work activities, and recreational activities. Smoking status was classified into three groups: never (less than 100 cigarettes), former (≥100 cigarettes but quit), and now (≥100 cigarettes and still smoking). Alcohol consumption was self-reported and categorized as never (less than 12 drinks in their lifetime), former (less than 12 drinks in the past year), and now (12 or more drinks in the past year). Hypertension was classified as a condition when the systolic blood pressure reached ≥140 mmHg or the diastolic blood pressure was ≥90 mmHg. The criteria for diagnosing diabetes mellitus (DM) include a physician’s diagnosis, a glycohemoglobin (HbA1c) level greater than 6.5%, fasting blood glucose levels of 7.0 mmol/L or higher, random or two-hour oral glucose tolerance test (OGTT) results showing blood glucose levels of 11.1 mmol/L or above, or the use of diabetes medications or insulin. Hyperlipidemia was identified in individuals who met any of the following thresholds: (1) total cholesterol (TC) ≥ 200 mg/dL, (2) triglycerides (TG) ≥ 150 mg/dL, (3) low-density lipoprotein cholesterol (LDL-C) ≥ 130 mg/dL, or (4) high-density lipoprotein cholesterol (HDL-C) ≤ 40 mg/dL in men or ≤ 50 mg/dL in women. Chronic kidney disease (CKD) was defined by a urinary albumin-to-creatinine ratio of ≥30 mg/g or 3 mg/mmol, and/or an estimated glomerular filtration rate (eGFR) of <60 ml/min/1.73 m². Sitting time referred to the participant’s self-reported daily sedentary duration (in minutes). Sleep time represented the participant’s self-reported daily sleep duration (in hours). A family history of heart disease was assessed by asking participants, “including living and deceased, were any of your close biological that is, blood relatives including father, mother, sisters or brothers, ever told by a health professional that they had a heart attack or angina before the age of 50?” Chest pain was determined by asking participants, “ever had any pain or discomfort in your chest?” Participants took part in two 24-hour dietary recall interviews conducted at a mobile testing center, with a gap of 3-10 days between each interview. The data collected was used to calculate the average daily intake of various dietary nutrients, including macronutrients, minerals, vitamins, and others.

**Supplementary Methodology 2. Rationale for not using NHANES sampling weights in machine learning modeling.**

In the machine learning prediction component of our study, we did not apply NHANES sampling weights, primarily for the following reasons:

NHANES weights are designed to facilitate population-level inference, such as estimating national prevalence or means. However, the primary objective of our study was to develop an individual-level CVD risk prediction model, aimed at identifying high-risk individuals among patients with kidney stones. For this type of individual-level prediction task, it is widely accepted in the literature—and supported by several prior NHANES-based studies^1-4^ —that using unweighted data is both acceptable and common practice. Notably, NHANES official guidelines do not mandate the use of weights in predictive modeling.

Moreover, applying weights in machine learning may lead to overfitting to individuals with disproportionately high weights, thereby reducing the model’s generalizability and stability. From a technical perspective, the use of weights can also interfere with resampling techniques such as SMOTE, where the oversampling of the minority class may be distorted due to weight dilution. Additionally, most widely used machine learning algorithms (e.g., random forests, k-nearest neighbors) do not have standardized or consistent implementations for handling weighted data during model training.

For these reasons, we chose not to use survey weights in our modeling process, in order to better align with the goal of individual-level risk prediction while maintaining model stability and performance.

References:

1. Bai Q, Chen H, Gao Z, et al. Advanced Prediction of Heart Failure Risk in Elderly Diabetic and Hypertensive Patients Using Nine Machine Learning Models and Novel Composite Indices: Insights from NHANES 2003-2016. *Eur J Prev Cardiol*. Published online February 27, 2025:zwaf081. doi:10.1093/eurjpc/zwaf081

2. Qi X, Wang S, Fang C, Jia J, Lin L, Yuan T. Machine learning and SHAP value interpretation for predicting comorbidity of cardiovascular disease and cancer with dietary antioxidants. *Redox Biol*. 2025;79:103470. doi:10.1016/j.redox.2024.103470

3. Zhu G, Song Y, Lu Z, et al. Machine learning models for predicting metabolic dysfunction-associated steatotic liver disease prevalence using basic demographic and clinical characteristics. *J Transl Med*. 2025;23(1):381. doi:10.1186/s12967-025-06387-5

4. Lu X, Kou H, Li C, et al. Development and validation of an interpretable machine learning model for predicting hyperuricemia risk: Based on environmental chemical exposure. *Ecotoxicol Environ Saf*. 2025;299:118392. doi:10.1016/j.ecoenv.2025.118392

**Supplementary Methodology 3. Feature Selection Strategy and Implementation.**

In this study, feature selection was performed once on the entire development cohort (NHANES 2007–2016), rather than being repeated within each training fold of the nested cross-validation framework. Specifically, Boruta and LASSO were first applied independently to the full development dataset for feature selection. The intersection of features identified by both methods was then retained, and further refined based on clinical relevance, resulting in a final set of 15 predictors.

These selected features were subsequently fixed and used as inputs for model development. Nested cross-validation was then conducted within the same development cohort, with the inner loop used for hyperparameter tuning and the outer loop for performance evaluation. No additional feature selection was performed during this process.

**Supplementary Methodology 4. Handling class imbalance with SMOTE.**

To address class imbalance, we applied the SMOTE, which generates synthetic samples for the minority class by interpolating between neighboring minority instances. This approach enhances the model’s ability to learn minority class characteristics and promotes a more balanced representation of class features when defining decision boundaries. Our implementation adheres to the core principles of SMOTE, supplemented by nested resampling and hyperparameter tuning to ensure model robustness.

*Principle of SMOTE:*

SMOTE identifies nearest neighbors among minority-class samples and creates synthetic instances through interpolation. This increases sample diversity, enriches the training data, and reduces the risk of overfitting due to class imbalance.

*Application Strategy:*

In accordance with best practices, SMOTE was applied exclusively to the training set, preserving the original class distribution in the test set. We adopted a nested resampling strategy: the inner loop for hyperparameter optimization and the outer loop for model evaluation. This design ensures that model performance is assessed on data that reflects real-world distributions.

*Implementation in mlr3:*

We utilized the mlr3 framework’s graph learner architecture, combining the SMOTE module with various classifiers (e.g., Naive Bayes, Random Forest, RPART). This modular design enables learner-specific tuning of SMOTE parameters, improving performance while maintaining reproducibility and methodological rigor. For implementation details, refer to the official mlr3 tutorial[, Introduction and Overview – Applied Machine Learning Using mlr3 in R](https://mlr3book.mlr-org.com/chapters/chapter1/introduction_and_overview.html).

**Supplementary Discussion 1. Explanation for selecting AUC as the primary metric and the clinical implications of various indicators.**

We selected the area under the AUC as the primary evaluation metric due to its superior ability to assess model discrimination, which aligns closely with our objective of identifying high-risk CVD individuals among patients with kidney stones. AUC reflects the model's capacity to distinguish between high- and low-risk individuals across varying thresholds, making it especially suitable for early-stage risk screening. A higher AUC indicates better discriminative performance, supporting clinical decision-making and facilitating timely intervention. In contrast, the Brier score primarily evaluates calibration—the agreement between predicted probabilities and actual outcomes—and does not directly capture discriminative power. While calibration is important, discrimination is prioritized during initial screening. Additionally, AUC is robust to class imbalance, making it appropriate for datasets with low CVD prevalence. The use of AUC is consistent with prior studies, including those by Qi et al.,^1^ Zhu et al.,^2^ and Lu et al..^3^

In model selection, factors such as computational complexity and runtime efficiency were not prioritized as primary criteria because the study was conducted on a moderate-sized dataset (NHANES) and all evaluated algorithms are computationally tractable for offline analysis. Moreover, the main objective of this study was to identify the model with the best predictive performance rather than to optimize real-time deployment or resource-constrained implementation. Therefore, model selection was primarily driven by discrimination ability and overall predictive performance rather than computational burden.

In the study, we conducted a multi-dimensional evaluation of model performance. In the internal test set, the LR model achieved an AUC of 0.801, demonstrating strong discrimination. Sensitivity and specificity were 0.721 and 0.771, respectively, indicating effective identification of CVD cases while minimizing false positives. The model’s accuracy was 0.759, and balanced accuracy was 0.746, supporting its suitability for practical screening applications. The Brier score was 0.169, reflecting good calibration, and the F1 score was 0.590, showing a reasonable balance between precision and recall. Although the F1 score suggests room for improvement, it remains informative, especially in imbalanced settings. Clinically, high sensitivity and specificity enhance resource allocation and risk management, while good calibration builds trust in model predictions. Future work will focus on refining the model structure to further improve the F1 score and enhance diagnostic efficiency. We acknowledge the significant impact that false positive and false negative results can have on clinical practice. False positives may cause unnecessary patient anxiety, additional medical examinations, increased costs, and inefficient use of medical resources. Conversely, false negatives can lead to missed CVD diagnoses, delayed treatment, and greater risk of adverse events, thereby endangering patient health. In this study, the logistic regression model balances sensitivity and specificity to mitigate these risks. Its high specificity (0.771) reduces false positives, minimizing patient anxiety and economic burden from unwarranted follow-ups. Simultaneously, its high sensitivity (0.721) decreases false negatives, facilitating timely identification and intervention for most CVD cases. This balance is essential for effective clinical application, underscoring the model’s potential as a reliable CVD risk screening tool.

References:

1. Qi X, Wang S, Fang C, Jia J, Lin L, Yuan T. Machine learning and SHAP value interpretation for predicting comorbidity of cardiovascular disease and cancer with dietary antioxidants. *Redox Biol*. 2025;79:103470. doi:10.1016/j.redox.2024.103470

2. Zhu G, Song Y, Lu Z, et al. Machine learning models for predicting metabolic dysfunction-associated steatotic liver disease prevalence using basic demographic and clinical characteristics. *J Transl Med*. 2025;23(1):381. doi:10.1186/s12967-025-06387-5

3. Lu X, Kou H, Li C, et al. Development and validation of an interpretable machine learning model for predicting hyperuricemia risk: Based on environmental chemical exposure. *Ecotoxicol Environ Saf*. 2025;299:118392. doi:10.1016/j.ecoenv.2025.118392

**Supplementary Discussion 2. Justification for developing a dedicated model for identifying prevalent CVD in patients with kidney stone disease.**

Previous studies have established a significant association between kidney stones and CVD, with patients frequently exhibiting comorbidities such as metabolic syndrome, CKD, and DM, which collectively elevate CVD risk.^1-4^ Our analysis of NHANES data further indicates that individuals with kidney stones have a 47% higher adjusted risk of CVD compared to those without (adjusted OR = 1.47). The distinct pathophysiological characteristics and risk profiles of these patients, combined with lifestyle and dietary factors—such as dietary patterns, physical inactivity, and smoking—contribute to an increased cardiovascular burden.^5^ Consequently, a risk prediction model tailored to these unique factors could more accurately assess cardiovascular health in this population.

Our proposed model incorporates non-invasive, multidimensional variables including demographic factors, lifestyle behaviors, and dietary nutrient intake. These determinants not only influence kidney stone formation but also are critical regulators of CVD risk, yet they are often underrepresented in traditional predictive models. Our LR model achieved an AUC of 0.801 in the kidney stone cohort, surpassing the Q-Lite model’s AUC of 0.781. Although this difference was not statistically significant (DeLong test P = 0.234, Supplementary Table 9), the inclusion of lifestyle and nutritional variables enhances convenience and applicability for intervention guidance. DCA demonstrated superior net clinical benefit of the LR model over the Q-Lite model (Supplementary Figure 4), while calibration curves confirmed the model’s predictive reliability (Supplementary Figure 3).

Clinically, the model’s reliance on non-invasive measures facilitates integration into routine outpatient and community health services, enabling rapid CVD risk screening among patients with kidney stones. This capability supports efficient identification of high-risk individuals and allows for tailored lifestyle interventions and monitoring, thereby promoting personalized preventive strategies and improved clinical outcomes. For patients flagged as high-risk, further specialized cardiovascular assessments are recommended to ensure accurate diagnosis and timely management while minimizing unnecessary medical expenditures and optimizing resource allocation.

In summary, given the unique risk profile of patients with kidney stone disease and the limitations of existing models, the development of a specialized model for identifying prevalent CVD is both scientifically warranted and of clinical relevance.

References:

1. Ferraro PM, Taylor EN, Eisner BH, et al. History of kidney stones and the risk of coronary heart disease. *JAMA*. 2013;310(4):408-415. doi:10.1001/jama.2013.8780

2. Liu Y, Li S, Zeng Z, et al. Kidney stones and cardiovascular risk: a meta-analysis of cohort studies. *Am J Kidney Dis Off J Natl Kidney Found*. 2014;64(3):402-410. doi:10.1053/j.ajkd.2014.03.017

3. Alexander RT, Hemmelgarn BR, Wiebe N, et al. Kidney stones and cardiovascular events: a cohort study. *Clin J Am Soc Nephrol CJASN*. 2014;9(3):506-512. doi:10.2215/CJN.04960513

4. Khan SR, Pearle MS, Robertson WG, et al. Kidney stones. *Nat Rev Dis Primer*. 2016;2:16008. doi:10.1038/nrdp.2016.8

5. Yang Q, Lin H, Zhang X, et al. Life’s Essential 8 and kidney stones in US adults: mediating roles of HDL and insulin resistance. *Minerva Urol Nephrol*. 2025;77(1):120-129. doi:10.23736/S2724-6051.24.05774-4

**Supplementary Discussion 3. Validation of self-reported CVD and potential self-reporting bias.**

A previous validation study assessed the accuracy of self-reported kidney stone diagnoses in the Health Professionals Follow-up Study (HPFS) by randomly selecting 60 male participants and reviewing their medical records. Medical record verification confirmed the diagnosis in 97% of the cases.^1^ Similarly, in the Nurses’ Health Study I (NHS I), 90 female participants who self-reported kidney stones were randomly selected, and a comprehensive review of their medical records was conducted. All but one case were confirmed, resulting in a 98% confirmation rate.^2^ These findings support the feasibility and reliability of using self-reported diagnoses in epidemiological studies. Following this approach, we used self-reported data to identify kidney stone patients in the NHANES dataset.

However, as NHANES is a de-identified dataset, it is unfortunately not possible to independently validate self-reported diagnoses. Like previous NHANES-based studies on kidney stones and CVD,^3,4^ our study also relied on self-reported diagnosis data. To further assess the reliability of self-reported CVD diagnoses, we utilized ICD-10-coded diagnoses derived from prescription medication data collected during the 2013–2018 NHANES cycles. Specifically, during the in-home SP interview, participants were asked whether they had taken any prescription medications in the past 30 days. Those who answered “yes” were requested to show the interviewer all medication containers used. Interviewers then entered the full product names from the containers into a computerized system, which matched the entries with a prescription drug database to identify exact or approximate matches. Using this approach, we identified 56 participants with CVD based on ICD-10 codes. Among them, 53 had also self-reported CVD, while only 3 had not, yielding a concordance rate of 95%. While this method has limitations, we believe it provides supportive evidence for the validity of self-reported CVD in NHANES.

Nonetheless, both kidney stone disease and CVD were defined based on self-reported data, which may introduce misclassification bias affecting both association estimates and model performance. Non-differential misclassification generally biases effect estimates toward the null and reduces discriminative ability, suggesting that the observed association and reported AUC (0.801) may be conservative estimates under ideal measurement conditions. However, differential misclassification related to healthcare-seeking behavior or symptom awareness may introduce unpredictable bias, potentially affecting both the magnitude of associations and apparent model performance. Although our limited validation using NHANES prescription drug records (N = 56) suggests reasonable accuracy for self-reported CVD among treated individuals (94.6%, 53/56), it cannot fully capture undiagnosed cases, untreated individuals, or subgroup-specific reporting differences. Such misclassification may also influence feature importance derived from SHAP values, particularly for variables related to healthcare utilization and socioeconomic status, potentially inflating or attenuating their apparent importance. Therefore, SHAP-based feature rankings should be interpreted as reflecting associations with self-reported prevalent CVD rather than causal or mechanistic importance, and validation in cohorts with adjudicated clinical outcomes is warranted.

References:

1. Taylor EN, Stampfer MJ, Curhan GC. Obesity, weight gain, and the risk of kidney stones. *JAMA*. 2005;293(4):455-462. doi:10.1001/jama.293.4.455

2. Curhan GC, Willett WC, Speizer FE, Spiegelman D, Stampfer MJ. Comparison of dietary calcium with supplemental calcium and other nutrients as factors affecting the risk for kidney stones in women. *Ann Intern Med*. 1997;126(7):497-504. doi:10.7326/0003-4819-126-7-199704010-00001

3. Scales CD, Smith AC, Hanley JM, Saigal CS, Urologic Diseases in America Project. Prevalence of kidney stones in the United States. *Eur Urol*. 2012;62(1):160-165. doi:10.1016/j.eururo.2012.03.052

4. Dang K, Wang X, Hu J, et al. The association between triglyceride-glucose index and its combination with obesity indicators and cardiovascular disease: NHANES 2003-2018. *Cardiovasc Diabetol*. 2024;23(1):8. doi:10.1186/s12933-023-02115-9

**Supplementary Figure 1. Feature selection with Boruta.**

**
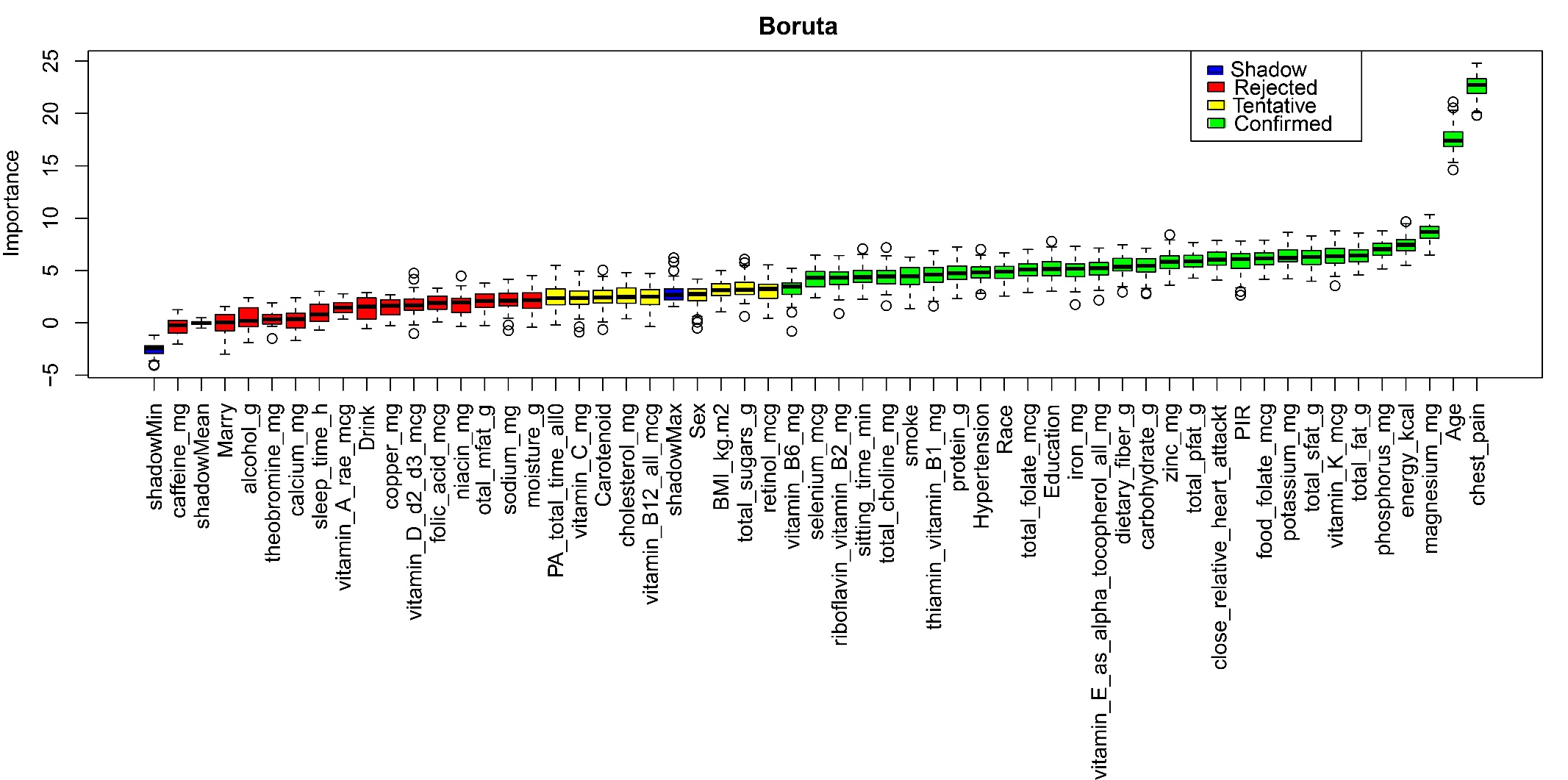
**

The blue bars represent the minimum, average, and maximum shadow feature importance scores. Variables shown with green box plots were confirmed as important, those in yellow were identified as tentative, and those in red were rejected.

**Supplementary Figure 2. Lasso regression-based variable screening.**

**
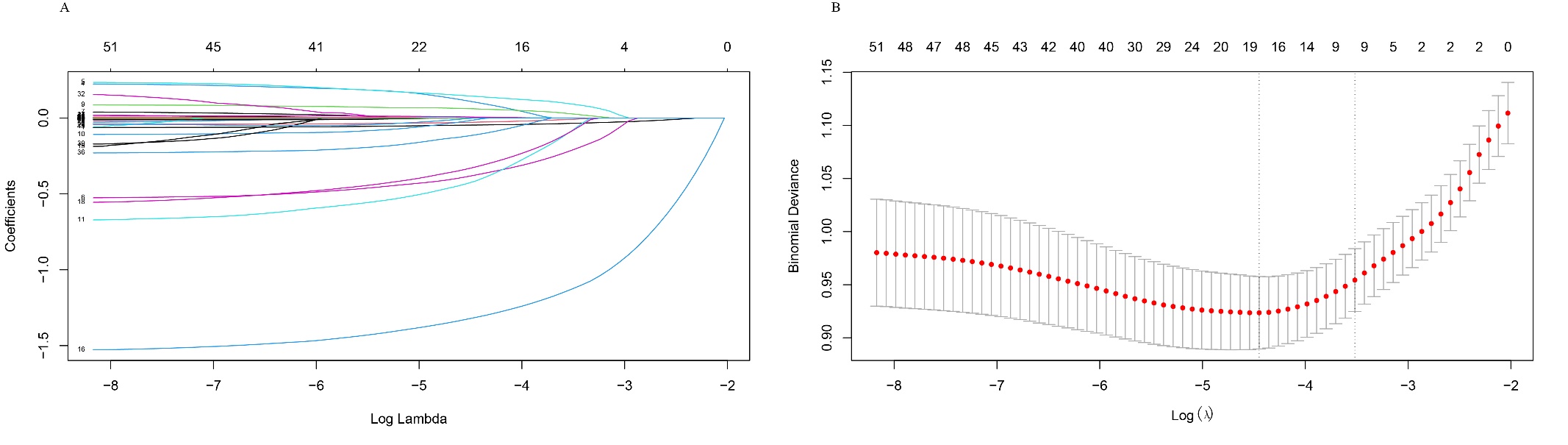
**

(A). Variation characteristics of variable coefficients. The 54 colored lines in the figure stand for 54 variables, which were selected through LASSO regression. The x-axis shows the log lambda, while the y-axis indicates the partial regression coefficient. As the logarithm of lambda increases, the regularization parameter becomes stronger, reducing the size of the regression coefficients and possibly shrinking them close to zero, which may lead to their removal from the model; (B). In the lasso regression model, cross-validation is used to find the best λ value. The dashed line on the left marks λ min, the lambda value at which the model has the best fit. The 15 variables corresponding to log (λ) min were chosen.

**Supplementary Figure 3. Calibration curve of LR model on internal test set.**


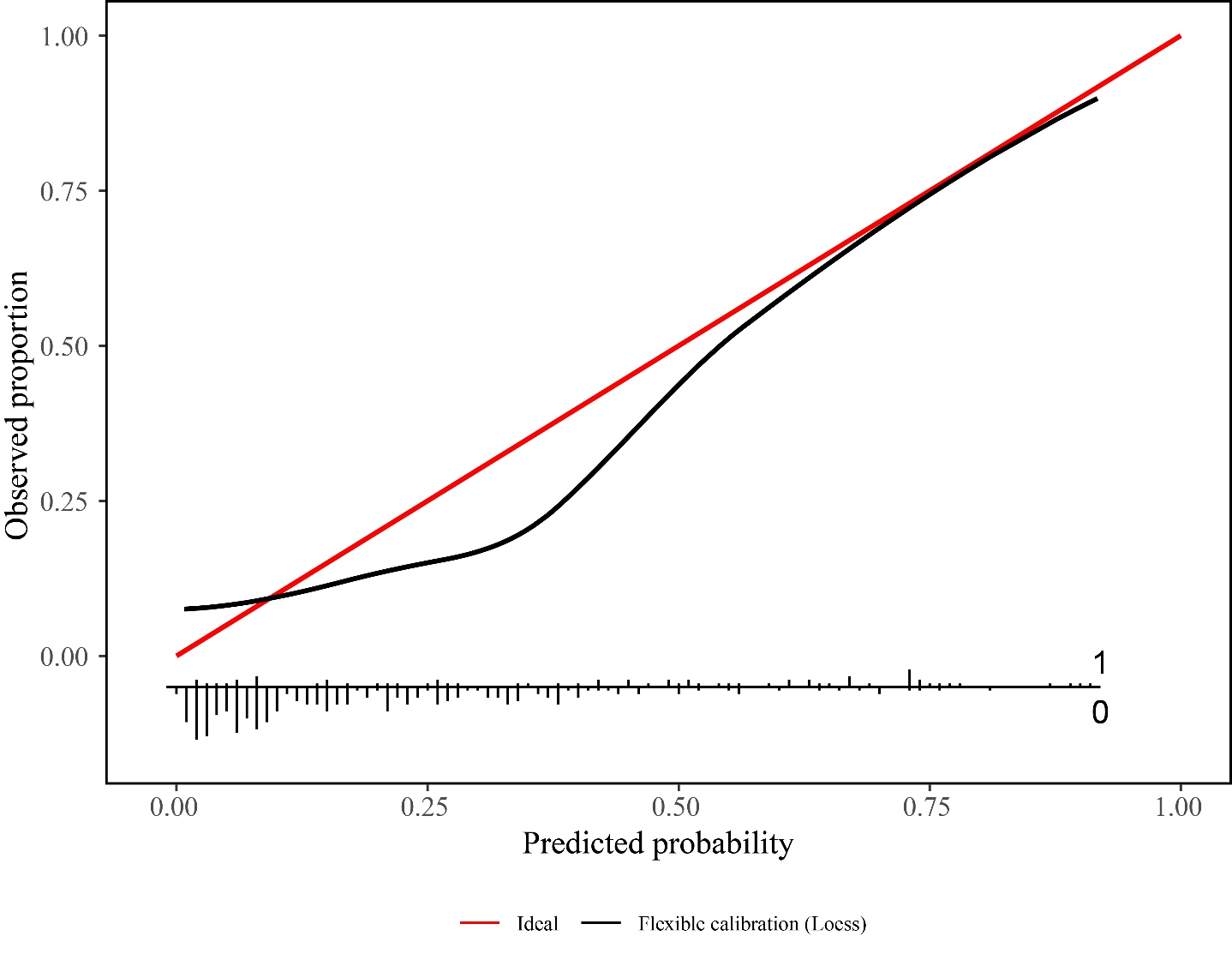


Abbreviations: LR, logistic regression.

**Supplementary Figure 4. Decision curve analysis for LR Model and Q-Lite model on internal test set.**


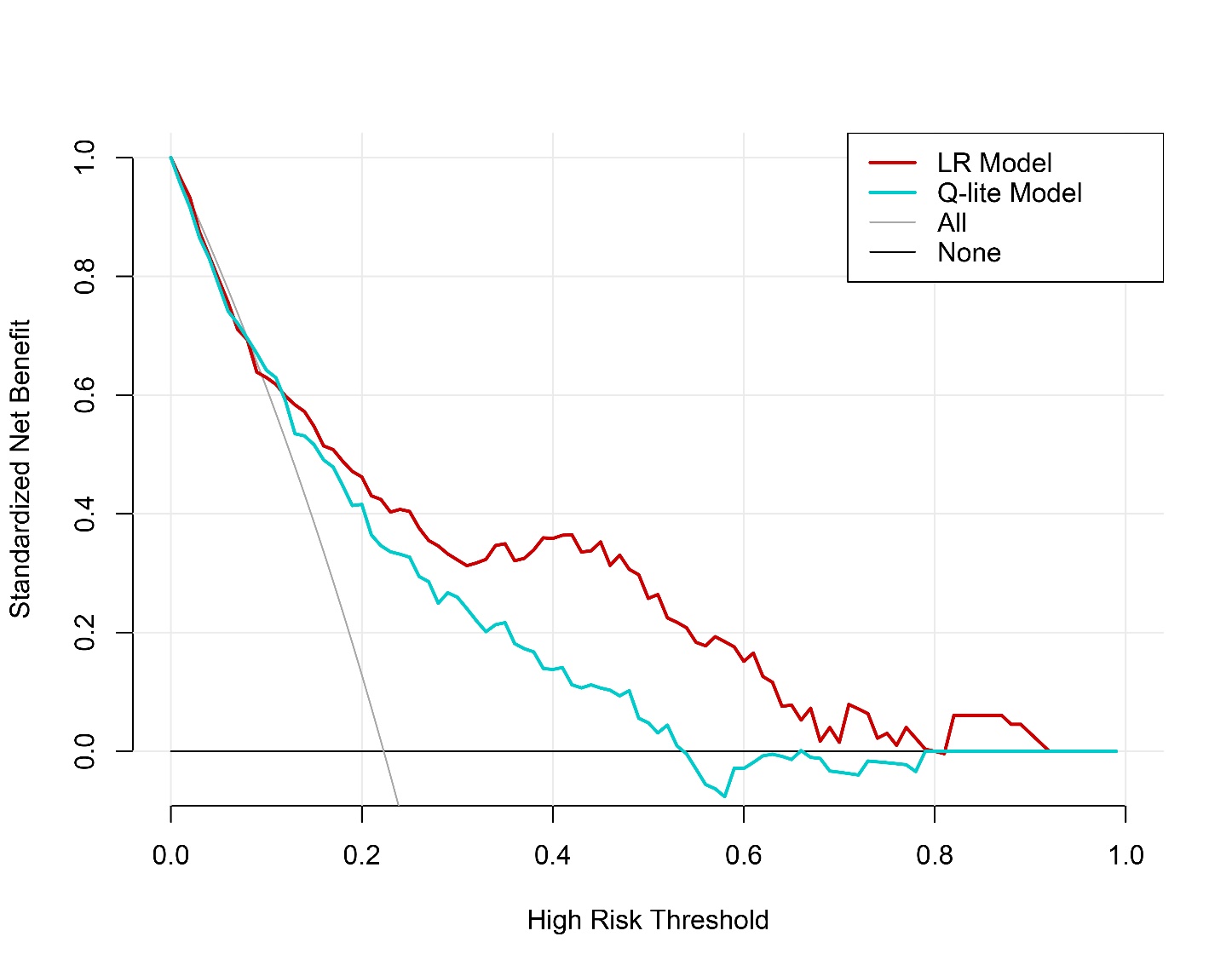


Abbreviations: LR, logistic regression.

**Supplementary Figure 5. Confusion matrix plot for LR model in the temporal validation cohort.**


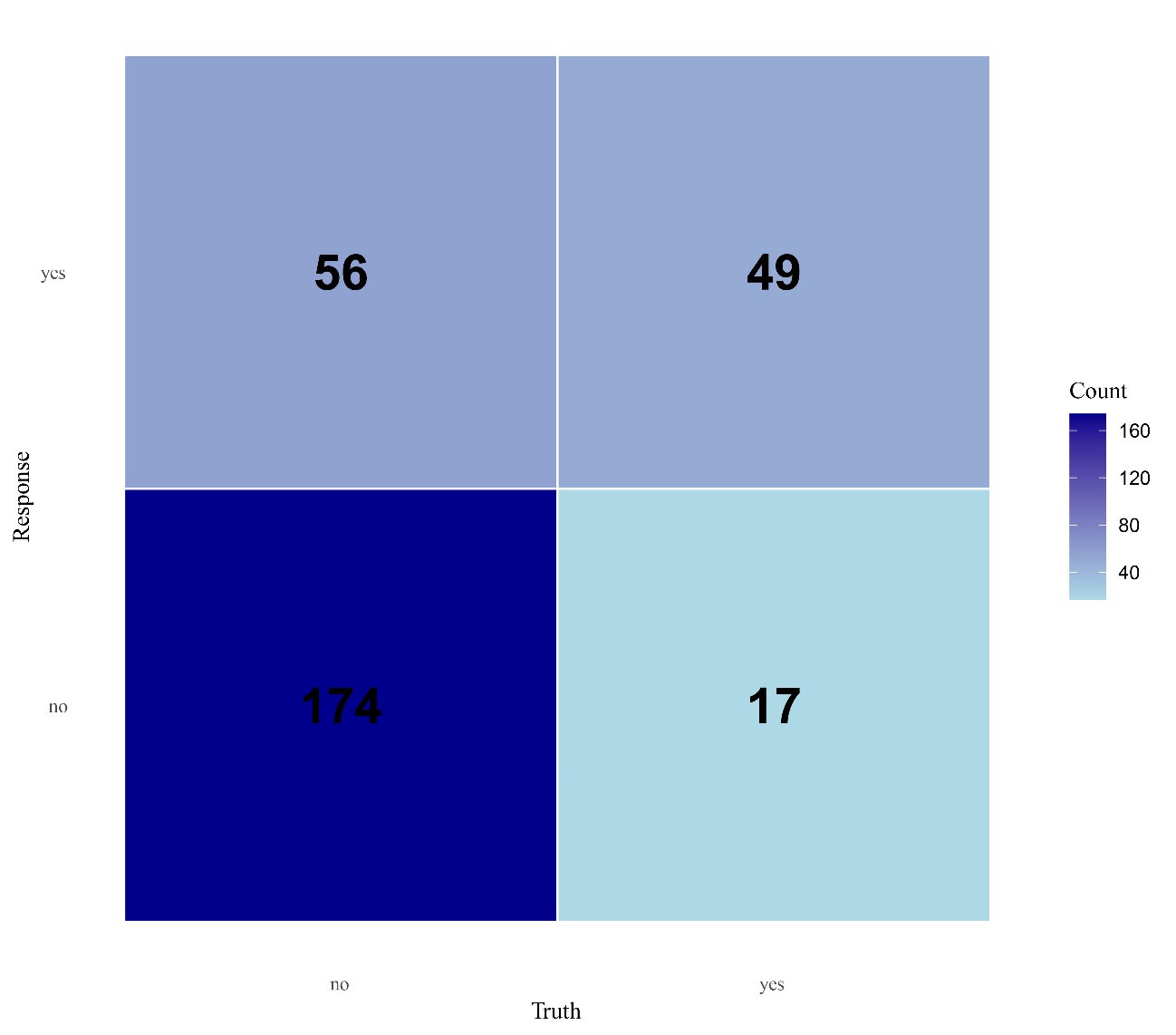


Abbreviations: LR, logistic regression.

**Supplementary Figure 6. Partial dependence plots.**

**
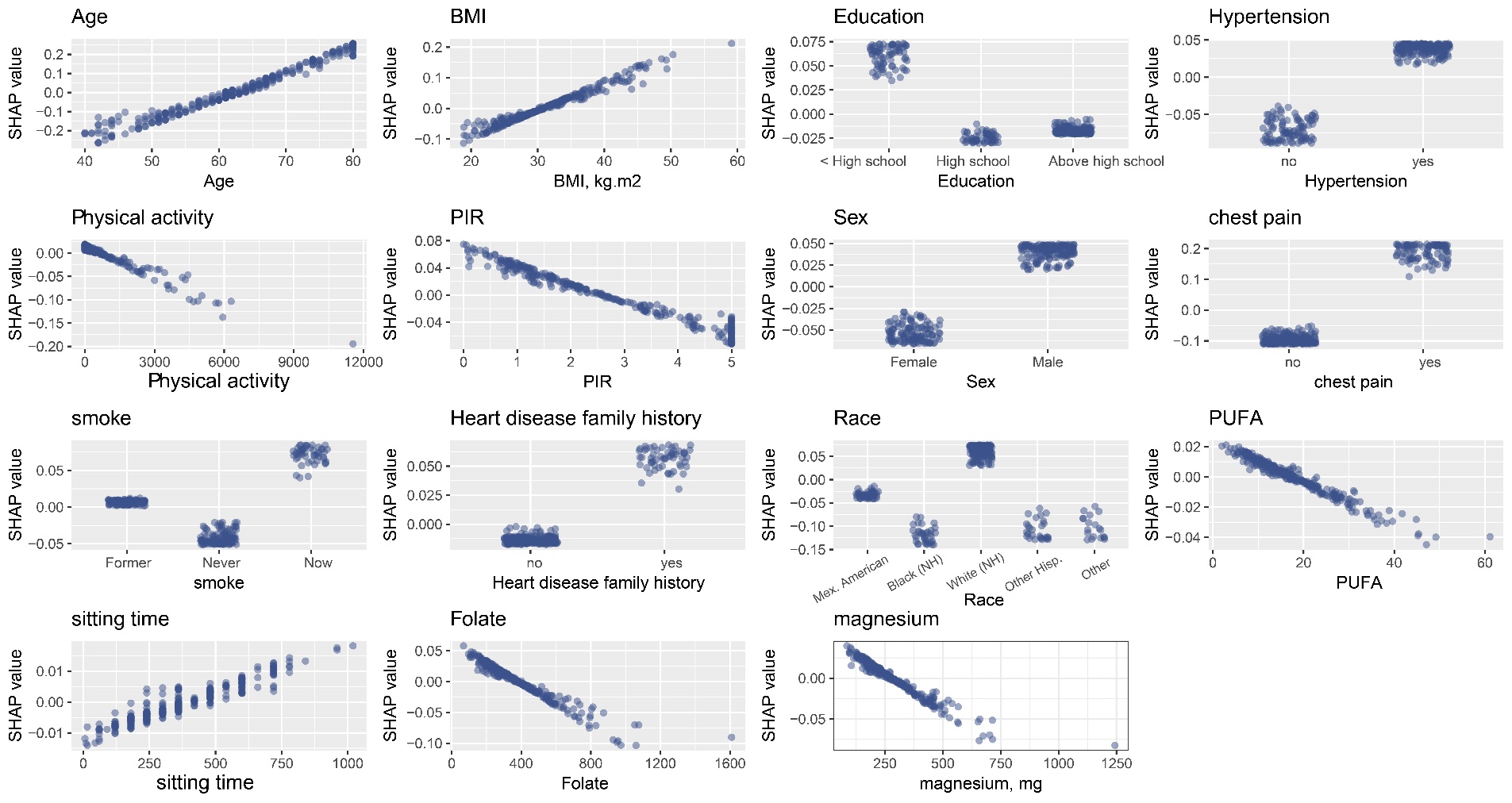
**

Abbreviations: BMI, body mass index; PIR, poverty income ratio; PUFA, polyunsaturated fatty acids. Education levels: < High school (less than high school), High school (high school or equivalent), above high school. Race categories: Mex. American (Mexican American), Black (NH) (non-Hispanic Black), White (NH) (non-Hispanic White), Other Hisp. (other Hispanic), Other (other race).

**Supplementary Table 1. Characteristics of patients with kidney stone disease in NHANES 2007 - 2018 cycles.**

| **Characteristic** | **Overall (N = 1,787)** | **Without CVD (N = 1,357)** | **With CVD (N = 430)** | ***P* value** |  |
| --- | --- | --- | --- | --- | --- |
| Age, years |  |  |  | <0.001 |  |
| Mean (SD) | 61.70 (11.85) | 59.93 (11.61) | 67.30 (10.83) |  |  |
| Median (Q1, Q3) | 62.00 (52, 71) | 60.00 (51, 68) | 68 (61, 77) |  |  |
| Sex, n (%) |  |  |  | 0.002 |  |
| Female | 750 (41.97) | 597 (43.99) | 153 (35.58) |  |  |
| Male | 1,037 (58.03) | 760 (56.01) | 277 (64.42) |  |  |
| Race, n (%) |  |  |  | 0.017 |  |
| Mexican American | 220 (12.31) | 177 (13.04) | 43 (10.00) |  |  |
| Non-Hispanic Black | 244 (13.65) | 195 (14.37) | 49 (11.40) |  |  |
| Non-Hispanic White | 1,031 (57.69) | 753 (55.49) | 278 (64.65) |  |  |
| Other Hispanic | 174 (9.74) | 135 (9.95) | 39 (9.07) |  |  |
| Other Race | 118 (6.60) | 97 (7.15) | 21 (4.88) |  |  |
| Marital status, n (%) |  |  |  | 0.10 |  |
| Married | 1,085 (60.72) | 840 (61.90) | 245 (56.98) |  |  |
| Never married | 97 (5.43) | 78 (5.75) | 19 (4.42) |  |  |
| Living with partner | 81 (4.53) | 59 (4.35) | 22 (5.12) |  |  |
| Other | 524 (29.32) | 380 (28.00) | 144 (33.49) |  |  |
| PIR |  |  |  | <0.001 |  |
| Mean (SD) | 2.64 (1.60) | 2.76 (1.62) | 2.24 (1.47) |  |  |
| Median (Q1, Q3) | 2.18 (1.24, 4.28) | 2.40 (1.31, 4.62) | 1.82 (1.06, 3.28) |  |  |
| Education level, n (%) |  |  |  | <0.001 |  |
| Less than high school | 398 (22.27) | 267 (19.68) | 131 (30.47) |  |  |
| High school or equivalent | 417 (23.34) | 317 (23.36) | 100 (23.26) |  |  |
| Above high school | 972 (54.39) | 773 (56.96) | 199 (46.28) |  |  |
| smoke, n (%) |  |  |  | <0.001 |  |
| Never | 852 (47.68) | 684 (50.41) | 168 (39.07) |  |  |
| Former | 647 (36.21) | 465 (34.27) | 182 (42.33) |  |  |
| Now | 288 (16.12) | 208 (15.33) | 80 (18.60) |  |  |
| Alcohol use, n (%) |  |  |  | <0.001 |  |
| Never | 237 (13.26) | 176 (12.97) | 61 (14.19) |  |  |
| Former | 488 (27.31) | 320 (23.58) | 168 (39.07) |  |  |
| Now | 1,062 (59.43) | 861 (63.45) | 201 (46.74) |  |  |
| Physical activity, min/week |  |  |  | <0.001 |  |
| Mean (SD) | 771.50 (1,470.90) | 853.93 (1,543.86) | 511.34 (1,176.47) |  |  |
| Median (Q1, Q3) | 175.00 (0.00, 780.00) | 210.00 (0.00, 900.00) | 60.00 (0.00, 480.00) |  |  |
| Sitting time, min/day |  |  |  | <0.001 |  |
| Mean (SD) | 395.74 (548.70) | 372.85 (421.16) | 467.96 (828.18) |  |  |
| Median (Q1, Q3) | 360.00 (240.00, 480.00) | 360.00 (180.00, 480.00) | 360.00 (240.00, 480.00) |  |  |
| Sleep time, h/day |  |  |  | >0.99 |  |
| Mean (SD) | 7.08 (2.68) | 7.03 (1.47) | 7.24 (4.79) |  |  |
| Median (Q1, Q3) | 7.00 (6.00, 8.00) | 7.00 (6.00, 8.00) | 7.00 (6.00, 8.00) |  |  |
| BMI, kg/m^2^ |  |  |  | 0.002 |  |
| Mean (SD) | 30.83 (6.70) | 30.53 (6.50) | 31.79 (7.23) |  |  |
| Median (Q1, Q3) | 29.70 (26.10, 34.10) | 29.40 (25.90, 33.90) | 30.63 (26.70, 34.70) |  |  |
| Hypertension, n (%) |  |  |  | <0.001 |  |
| No | 630 (35.25) | 542 (39.94) | 88 (20.47) |  |  |
| Yes | 1,157 (64.75) | 815 (60.06) | 342 (79.53) |  |  |
| Chest pain, n (%) |  |  |  | <0.001 |  |
| No | 1,169 (65.42) | 1,002 (73.84) | 167 (38.84) |  |  |
| Yes | 618 (34.58) | 355 (26.16) | 263 (61.16) |  |  |
| Heart disease family history, n (%) |  |  |  | <0.001 |  |
| No | 1,435 (80.30) | 1,126 (82.98) | 309 (71.86) |  |  |
| Yes | 352 (19.70) | 231 (17.02) | 121 (28.14) |  |  |
| Energy intake, kcal/d |  |  |  | <0.001 |  |
| Mean (SD) | 1,972.62 (766.43) | 2,015.19 (767.98) | 1,838.28 (746.61) |  |  |
| Median (Q1, Q3) | 1,860.50 (1,438.00, 2,381.00) | 1,891.50 (1,485.50, 2,426.50) | 1,768.50 (1,285.50, 2,201.00) |  |  |
| Protein, g |  |  |  | <0.001 |  |
| Mean (SD) | 77.04 (32.87) | 78.47 (33.00) | 72.50 (32.06) |  |  |
| Median (Q1, Q3) | 71.86 (53.90, 93.61) | 73.25 (55.16, 96.16) | 67.82 (50.07, 89.19) |  |  |
| Carbohydrate, g |  |  |  | <0.001 |  |
| Mean (SD) | 237.13 (100.11) | 242.16 (100.47) | 221.25 (97.39) |  |  |
| Median (Q1, Q3) | 221.94 (166.18, 290.83) | 226.90 (170.48, 297.21) | 208.51 (154.69, 274.61) |  |  |
| Sugars, g |  |  |  | 0.008 |  |
| Mean (SD) | 104.11 (62.62) | 105.67 (62.13) | 99.22 (63.98) |  |  |
| Median (Q1, Q3) | 91.78 (61.98, 132.54) | 94.09 (63.65, 134.24) | 84.58 (55.07, 127.16) |  |  |
| Dietary fiber, g |  |  |  | 0.003 |  |
| Mean (SD) | 16.60 (9.16) | 17.03 (9.52) | 15.23 (7.79) |  |  |
| Median (Q1, Q3) | 15.00 (10.35, 21.20) | 15.45 (10.50, 21.60) | 13.85 (10.15, 19.75) |  |  |
| Fat, g |  |  |  | <0.001 |  |
| Mean (SD) | 78.25 (37.17) | 80.00 (37.38) | 72.71 (35.99) |  |  |
| Median (Q1, Q3) | 72.58 (52.67, 97.15) | 74.25 (53.94, 99.35) | 67.31 (47.35, 89.06) |  |  |
| Saturated fatty acids, g |  |  |  | 0.002 |  |
| Mean (SD) | 25.22 (12.95) | 25.65 (12.94) | 23.85 (12.90) |  |  |
| Median (Q1, Q3) | 22.84 (16.29, 31.72) | 23.17 (16.75, 32.06) | 21.59 (14.76, 29.48) |  |  |
| Monounsaturated fatty acids, g |  |  |  | <0.001 |  |
| Mean (SD) | 27.99 (14.21) | 28.61 (14.41) | 26.03 (13.37) |  |  |
| Median (Q1, Q3) | 25.63 (18.32, 35.03) | 26.11 (18.86, 35.69) | 24.22 (17.04, 32.36) |  |  |
| PUFA, g |  |  |  | <0.001 |  |
| Mean (SD) | 17.97 (9.67) | 18.50 (9.71) | 16.28 (9.35) |  |  |
| Median (Q1, Q3) | 16.25 (11.04, 22.53) | 16.64 (11.51, 23.31) | 14.87 (9.98, 19.94) |  |  |
| Cholesterol, mg |  |  |  | 0.31 |  |
| Mean (SD) | 295.49 (192.02) | 298.05 (194.25) | 287.43 (184.80) |  |  |
| Median (Q1, Q3) | 251.50 (156.00, 389.00) | 254.00 (156.50, 392.00) | 243.00 (155.50, 379.50) |  |  |
| Vitamin A rae, mcg |  |  |  | 0.30 |  |
| Mean (SD) | 618.01 (493.10) | 619.13 (462.61) | 614.49 (579.49) |  |  |
| Median (Q1, Q3) | 520.50 (324.50, 789.50) | 527.00 (330.00, 789.50) | 506.75 (313.00, 785.50) |  |  |
| Retinol, mcg |  |  |  | 0.45 |  |
| Mean (SD) | 423.95 (415.62) | 421.74 (374.20) | 430.94 (525.81) |  |  |
| Median (Q1, Q3) | 352.00 (211.50, 535.00) | 353.50 (213.00, 541.00) | 351.50 (207.50, 500.50) |  |  |
| Carotenoid |  |  |  | 0.029 |  |
| Mean (SD) | 8,746.44 (9,030.91) | 8,963.92 (9,234.80) | 8,060.13 (8,327.91) |  |  |
| Median (Q1, Q3) | 5,914.00 (2,851.00, 11,580.00) | 6,035.00 (2,958.50, 11,900.00) | 5,346.50 (2,385.50, 10,676.50) |  |  |
| Thiamin (vitamin B1), mg |  |  |  | 0.010 |  |
| Mean (SD) | 1.56 (0.70) | 1.58 (0.72) | 1.48 (0.65) |  |  |
| Median (Q1, Q3) | 1.43 (1.08, 1.91) | 1.44 (1.09, 1.93) | 1.37 (1.03, 1.87) |  |  |
| Riboflavin (vitamin B2), mg |  |  |  | 0.017 |  |
| Mean (SD) | 2.02 (0.94) | 2.05 (0.96) | 1.92 (0.86) |  |  |
| Median (Q1, Q3) | 1.86 (1.39, 2.49) | 1.90 (1.40, 2.52) | 1.80 (1.32, 2.34) |  |  |
| Niacin, mg |  |  |  | <0.001 |  |
| Mean (SD) | 23.86 (11.80) | 24.47 (12.15) | 21.90 (10.42) |  |  |
| Median (Q1, Q3) | 21.78 (16.00, 29.22) | 22.28 (16.28, 29.81) | 19.81 (14.88, 26.94) |  |  |
| Vitamin B6, mg |  |  |  | 0.002 |  |
| Mean (SD) | 1.96 (1.14) | 2.01 (1.20) | 1.80 (0.90) |  |  |
| Median (Q1, Q3) | 1.73 (1.26, 2.41) | 1.77 (1.30, 2.44) | 1.63 (1.19, 2.25) |  |  |
| Folate, mcg |  |  |  | 0.002 |  |
| Mean (SD) | 380.68 (197.31) | 389.39 (201.97) | 353.19 (179.29) |  |  |
| Median (Q1, Q3) | 342.50 (248.50, 472.50) | 348.00 (253.00, 484.00) | 324.25 (229.00, 450.50) |  |  |
| Folic acid, mcg |  |  |  | 0.11 |  |
| Mean (SD) | 173.28 (140.88) | 175.76 (142.29) | 165.49 (136.21) |  |  |
| Median (Q1, Q3) | 136.00 (83.00, 225.50) | 137.00 (85.00, 227.50) | 131.00 (75.50, 218.00) |  |  |
| Food folate, mcg |  |  |  | <0.001 |  |
| Mean (SD) | 207.45 (113.80) | 213.66 (118.65) | 187.85 (94.36) |  |  |
| Median (Q1, Q3) | 186.50 (132.50, 255.00) | 191.00 (134.50, 261.50) | 171.75 (127.00, 228.50) |  |  |
| Total choline, mg |  |  |  | 0.081 |  |
| Mean (SD) | 325.15 (154.10) | 328.42 (154.77) | 314.83 (151.70) |  |  |
| Median (Q1, Q3) | 296.90 (216.70, 404.50) | 298.60 (218.60, 408.00) | 290.80 (208.85, 390.05) |  |  |
| Vitamin B12, mcg |  |  |  | 0.94 |  |
| Mean (SD) | 5.90 (6.83) | 5.89 (6.78) | 5.91 (6.99) |  |  |
| Median (Q1, Q3) | 4.22 (2.54, 7.18) | 4.20 (2.51, 7.18) | 4.28 (2.60, 7.15) |  |  |
| Vitamin C, mg |  |  |  | 0.13 |  |
| Mean (SD) | 79.57 (75.74) | 81.20 (77.88) | 74.43 (68.38) |  |  |
| Median (Q1, Q3) | 58.40 (29.10, 108.25) | 61.50 (28.90, 111.30) | 52.83 (30.10, 98.25) |  |  |
| Vitamin D (D2+D3), mcg |  |  |  | 0.43 |  |
| Mean (SD) | 4.61 (4.21) | 4.61 (4.31) | 4.59 (3.89) |  |  |
| Median (Q1, Q3) | 3.55 (1.95, 5.95) | 3.55 (1.90, 5.95) | 3.73 (2.10, 5.75) |  |  |
| Vitamin E as alpha tocopherol, mg |  |  |  | <0.001 |  |
| Mean (SD) | 8.89 (7.73) | 9.24 (8.24) | 7.79 (5.68) |  |  |
| Median (Q1, Q3) | 7.02 (4.82, 10.60) | 7.22 (4.98, 10.97) | 6.53 (4.40, 9.29) |  |  |
| Vitamin K, mcg |  |  |  | <0.001 |  |
| Mean (SD) | 109.95 (124.19) | 113.84 (128.73) | 97.66 (107.84) |  |  |
| Median (Q1, Q3) | 74.95 (45.05, 125.80) | 78.85 (46.95, 128.95) | 63.05 (40.50, 112.85) |  |  |
| Calcium, mg |  |  |  | <0.001 |  |
| Mean (SD) | 869.90 (435.00) | 885.07 (429.37) | 822.02 (449.45) |  |  |
| Median (Q1, Q3) | 794.00 (560.50, 1,096.00) | 815.00 (579.00, 1,112.00) | 741.75 (501.00, 1,052.50) |  |  |
| Phosphorus, mg |  |  |  | <0.001 |  |
| Mean (SD) | 1,282.56 (515.56) | 1,309.35 (518.17) | 1,198.00 (498.43) |  |  |
| Median (Q1, Q3) | 1,212.50 (920.50, 1,575.50) | 1,233.00 (948.00, 1,601.00) | 1,171.50 (853.50, 1,440.50) |  |  |
| Magnesium, mg |  |  |  | <0.001 |  |
| Mean (SD) | 282.80 (122.19) | 291.05 (125.64) | 256.76 (106.64) |  |  |
| Median (Q1, Q3) | 260.50 (196.00, 345.00) | 268.00 (200.00, 354.50) | 246.25 (184.50, 305.00) |  |  |
| Iron, mg |  |  |  | 0.25 |  |
| Mean (SD) | 14.44 (7.40) | 14.60 (7.54) | 13.93 (6.95) |  |  |
| Median (Q1, Q3) | 12.77 (9.62, 17.50) | 12.90 (9.67, 17.53) | 12.39 (9.46, 17.22) |  |  |
| Zinc, mg |  |  |  | 0.17 |  |
| Mean (SD) | 10.73 (5.50) | 10.83 (5.58) | 10.40 (5.23) |  |  |
| Median (Q1, Q3) | 9.66 (6.98, 13.42) | 9.69 (7.10, 13.51) | 9.61 (6.79, 12.99) |  |  |
| Copper, mg |  |  |  | <0.001 |  |
| Mean (SD) | 1.21 (0.78) | 1.23 (0.71) | 1.15 (0.96) |  |  |
| Median (Q1, Q3) | 1.08 (0.81, 1.45) | 1.10 (0.82, 1.49) | 1.03 (0.80, 1.33) |  |  |
| Sodium, mg |  |  |  | <0.001 |  |
| Mean (SD) | 3,291.74 (1,416.20) | 3,348.65 (1,411.45) | 3,112.14 (1,417.81) |  |  |
| Median (Q1, Q3) | 3,080.50 (2,315.00, 4,012.00) | 3,116.50 (2,354.00, 4,071.50) | 2,908.00 (2,173.50, 3,784.00) |  |  |
| Potassium, mg |  |  |  | 0.010 |  |
| Mean (SD) | 2,579.34 (1,031.23) | 2,623.32 (1,058.85) | 2,440.57 (926.27) |  |  |
| Median (Q1, Q3) | 2,468.00 (1,854.50, 3,122.00) | 2,489.00 (1,886.50, 3,166.50) | 2,403.00 (1,785.50, 2,974.50) |  |  |
| Selenium, mcg |  |  |  | <0.001 |  |
| Mean (SD) | 108.07 (50.60) | 110.23 (50.76) | 101.27 (49.53) |  |  |
| Median (Q1, Q3) | 101.90 (73.15, 130.40) | 103.50 (74.80, 133.15) | 96.10 (68.45, 122.45) |  |  |
| Caffeine, mg |  |  |  | 0.24 |  |
| Mean (SD) | 164.34 (176.31) | 166.84 (177.87) | 156.42 (171.27) |  |  |
| Median (Q1, Q3) | 119.00 (43.00, 231.00) | 122.00 (44.00, 234.50) | 112.25 (40.00, 224.50) |  |  |
| Theobromine, mg |  |  |  | 0.21 |  |
| Mean (SD) | 33.02 (52.37) | 33.83 (52.91) | 30.45 (50.58) |  |  |
| Median (Q1, Q3) | 11.00 (0.00, 45.00) | 11.00 (0.00, 46.00) | 11.25 (0.00, 40.00) |  |  |
| Alcohol, g |  |  |  | 0.009 |  |
| Mean (SD) | 5.59 (15.01) | 5.82 (14.72) | 4.86 (15.87) |  |  |
| Median (Q1, Q3) | 0.00 (0.00, 0.00) | 0.00 (0.00, 0.75) | 0.00 (0.00, 0.00) |  |  |
| Moisture, g |  |  |  | <0.001 |  |
| Mean (SD) | 2,678.99 (1,161.16) | 2,717.46 (1,139.05) | 2,557.60 (1,221.79) |  |  |
| Median (Q1, Q3) | 2,477.89 (1,884.71, 3,240.06) | 2,516.32 (1,935.01, 3,288.38) | 2,342.21 (1,748.45, 3,049.48) |  |  |
| Abbreviations: BMI, body mass index; CVD, cardiovascular disease; M: Median; NHANES, National Health and Nutrition Examination Survey; PIR, poverty income ratio; Q, Quartile; rae, retinol activity equivalents; SD, Standard Deviation; PUFA, polyunsaturated fatty acids.  The Wilcoxon rank sum test compared continuous variables, and Pearson's Chi-squared test compared categorical variables for intergroup differences. Folate: This term represents the cumulative intake of Food folate, Folic acid and folic acid supplements; Food folate: refers exclusively to naturally occurring folic acid inherent in food; Folic acid: refers exclusively to synthetic folic acid added to food through mandatory food fortification programs. | | | | | |

**Supplementary Table 2. Characteristics of participants in the NHANES 2007-2018 cycles after propensity score matching.**

| **Characteristic** | **Without CVD (N = 2,411)** | **With CVD (N = 2,411)** | ***P* value** |
| --- | --- | --- | --- |
| Age, Mean (SD), years | 66.02 (12.60) | 66.05 (12.46) | 0.925 |
| Sex, n (%) |  |  |  |
| Female | 1,072 (44.5%) | 1,026 (42.6%) | 0.191 |
| Male | 1,339 (55.5%) | 1,385 (57.4%) |  |
| Race, n (%) |  |  |  |
| Mexican American | 198 (8.2%) | 197 (8.2%) | 0.550 |
| Non-Hispanic Black | 569 (23.6%) | 523 (21.7%) |  |
| Non-Hispanic White | 1,314 (54.5%) | 1,367 (56.7%) |  |
| Other Hispanic | 189 (7.8%) | 186 (7.7%) |  |
| Other Race | 141 (5.8%) | 138 (5.7%) |  |
| Marital status, n (%) |  |  |  |
| Married | 1,256 (52.1%) | 1,274 (52.8%) | 0.936 |
| Never married | 168 (7.0%) | 171 (7.1%) |  |
| Living with partner | 109 (4.5%) | 104 (4.3%) |  |
| Other | 878 (36.4%) | 862 (35.8%) |  |
| PIR, Mean (SD) | 2.24 (1.49) | 2.23 (1.49) | 0.932 |
| Education level, n (%) |  |  |  |
| Less than high school | 720 (29.9%) | 709 (29.4%) | 0.921 |
| High school or equivalent | 633 (26.3%) | 643 (26.7%) |  |
| Above high school | 1,058 (43.9%) | 1,059 (43.9%) |  |
| Energy intake, mean (SD), kcal/d | 1,820.74 (693.30) | 1,835.67 (756.28) | 0.475 |
| smoke, n (%) |  |  | 0.995 |
| Never | 934 (38.7%) | 931 (38.6%) |  |
| Former | 967 (40.1%) | 968 (40.1%) |  |
| Now | 510 (21.2%) | 512 (21.2%) |  |
| Alcohol use, n (%) |  |  | 0.580 |
| Never | 294 (12.2%) | 302 (12.5%) |  |
| Former | 789 (32.7%) | 817 (33.9%) |  |
| Now | 1,328 (55.1%) | 1,292 (53.6%) |  |
| Physical activity, Mean (SD), min/week | 543.56 (1125.71) | 534.31(1205.58) | 0.783 |
| BMI, Mean (SD), kg/m^2^ | 30.85 (7.30) | 30.81 (7.43) | 0.853 |
| Hypertension, n (%) |  |  | 0.494 |
| No | 472 (19.6%) | 492 (20.4%) |  |
| Yes | 1,939 (80.4%) | 1,919 (79.6%) |  |
| DM, n (%) |  |  | 0.542 |
| No | 1,343 (55.7%) | 1,365 (56.6%) |  |
| Yes | 1,068 (44.3%) | 1,046 (43.4%) |  |
| Hyperlipidemia, n (%) |  |  | 0.645 |
| No | 271 (11.2%) | 260 (10.8%) |  |
| Yes | 2,140 (88.8%) | 2,151 (89.2%) |  |
| CKD, n (%) |  |  | 0.284 |
| No | 1,350 (56.0%) | 1,312 (54.4%) |  |
| Yes | 1,061 (44.0%) | 1,099 (45.6%) |  |

Abbreviations: BMI, body mass index; CVD, cardiovascular disease; NHANES, National Health and Nutrition Examination Survey; PIR, poverty income ratio; SD, Standard Deviation.

**Supplementary Table 3. Association between kidney stones and CVD in different subgroups.**

| **Subgroup** | **Kidney stones** | **Case/Total** | | **OR(95%CI)** | ***P* value** | ***P* for interaction** |
| --- | --- | --- | --- | --- | --- | --- |
| **Age group** | | |  |  |  | 0.37 |
| 20-39 | No | 82/6,469 | | 1[Reference] |  |  |
|  | Yes | 14/395 | | 1.04(0.54, 2.01) | 0.90 |  |
| 40-59 | No | 427/6,535 | | 1[Reference] |  |  |
|  | Yes | 89/733 | | 1.80(1.26, 2.55) | 0.001 |  |
| ≥60 | No | 1,471/6,467 | | 1[Reference] |  |  |
|  | Yes | 328/1,033 | | 1.31(1.02, 1.68) | 0.03 |  |
| **Sex** |  |  | |  |  | 0.85 |
| Female | No | 874/1,0139 | | 1[Reference] |  |  |
|  | Yes | 152/934 | | 1.41(1.06, 1.88) | 0.02 |  |
| Male | No | 1,106/9,332 | | 1[Reference] |  |  |
|  | Yes | 279/1,227 | | 1.49(1.16, 1.90) | 0.002 |  |
| **Race** |  |  | |  |  | 0.11 |
| Mexican American | No | 155/2,770 | | 1[Reference] |  |  |
|  | Yes | 42/259 | | 1.90(1.18, 3.04) | 0.01 |  |
| Non-Hispanic Black | No | 472/4,142 | | 1[Reference] |  |  |
|  | Yes | 51/281 | | 1.01(0.71, 1.44) | 0.94 |  |
| Non-Hispanic White | No | 1,090/8,545 | | 1[Reference] |  |  |
|  | Yes | 277/1,257 | | 1.44(1.15, 1.80) | 0.002 |  |
| Other Hispanic | No | 146/1,895 | | 1[Reference] |  |  |
|  | Yes | 40/214 | | 2.09(1.21, 3.63) | 0.01 |  |
| Other Race | No | 117/2,119 | | 1[Reference] |  |  |
|  | Yes | 21/150 | | 1.82(0.90, 3.66) | 0.09 |  |
| **Marital status** |  |  | |  |  | 0.68 |
| Married | No | 1,029/10,076 | | 1[Reference] |  |  |
|  | Yes | 245/1,262 | | 1.38(1.08, 1.78) | 0.01 |  |
| Never married | No | 149/3,613 | | 1[Reference] |  |  |
|  | Yes | 22/198 | | 1.86(0.93, 3.72) | 0.08 |  |
| Living with partner | No | 79/1,563 | | 1[Reference] |  |  |
|  | Yes | 25/140 | | 2.82(1.02, 7.75) | 0.05 |  |
| Other | No | 723/4,219 | | 1[Reference] |  |  |
|  | Yes | 139/561 | | 1.39(1.01, 1.92) | 0.05 |  |
| **Education level** | | |  |  |  | 0.60 |
| Less than high school | No | 575/4,157 | | 1[Reference] |  |  |
|  | Yes | 134/481 | | 1.73(1.13, 2.64) | 0.01 |  |
| High school or equivalent | No | 546/4,473 | | 1[Reference] |  |  |
|  | Yes | 97/490 | | 1.26(0.91, 1.75) | 0.15 |  |
| Above high school | No | 859/10,841 | | 1[Reference] |  |  |
|  | Yes | 200/1,190 | | 1.50(1.11, 2.01) | 0.01 |  |
| **Alcohol use** |  |  | |  |  | 0.47 |
| Never | No | 242/2,555 | | 1[Reference] |  |  |
|  | Yes | 60/274 | | 1.79(1.14, 2.82) | 0.01 |  |
| Former | No | 656/3,463 | | 1[Reference] |  |  |
|  | Yes | 161/536 | | 1.56(1.13, 2.15) | 0.01 |  |
| Now | No | 1,082/13,453 | | 1[Reference] |  |  |
|  | Yes | 210/1,351 | | 1.35(1.04, 1.73) | 0.02 |  |
| **smoke** |  |  | |  |  | 0.57 |
| Never | No | 764/10,921 | | 1[Reference] |  |  |
|  | Yes | 167/1,039 | | 1.64(1.24, 2.17) | <0.001 |  |
| Former | No | 786/4,752 | | 1[Reference] |  |  |
|  | Yes | 182/704 | | 1.31(0.97, 1.77) | 0.08 |  |
| Now | No | 430/3,798 | | 1[Reference] |  |  |
|  | Yes | 82/418 | | 1.47(0.96, 2.26) | 0.08 |  |
| **Hypertension** | | |  |  |  | 0.21 |
| No | No | 399/11,274 | | 1[Reference] |  |  |
|  | Yes | 93/916 | | 1.66(1.18, 2.34) | 0.004 |  |
| Yes | No | 1,581/8,197 | | 1[Reference] |  |  |
|  | Yes | 338/1,245 | | 1.38(1.10, 1.72) | 0.01 |  |
| **DM** |  |  | |  |  | **0.04** |
| No | No | 1,163/15,938 | | 1[Reference] |  |  |
|  | Yes | 202/1,510 | | 1.25(0.98, 1.61) | 0.08 |  |
| Yes | No | 817/3,533 | | 1[Reference] |  |  |
|  | Yes | 229/651 | | 1.85(1.40, 2.45) | <0.001 |  |
| **Hyperlipidemia** | | |  |  |  | 0.66 |
| No | No | 223/5,540 | | 1[Reference] |  |  |
|  | Yes | 37/441 | | 1.73(1.00, 2.96) | 0.05 |  |
| Yes | No | 1,757/13,931 | | 1[Reference] |  |  |
|  | Yes | 394/1,720 | | 1.47(1.18, 1.84) | <0.001 |  |
| **CKD** |  |  | |  |  | 0.32 |
| No | No | 1,092/16,070 | | 1[Reference] |  |  |
|  | Yes | 220/1,573 | | 1.54(1.20, 1.98) | <0.001 |  |
| Yes | No | 888/3,401 | | 1[Reference] |  |  |
|  | Yes | 211/588 | | 1.32(0.99, 1.78) | 0.06 |  |

Abbreviations: BMI, body mass index; CKD, chronic kidney disease; CI, Confidence Interval; CVD, cardiovascular disease; DM, diabetes mellitus; OR, Odd Ratio; PIR, poverty income ratio. The model was adjusted for age, sex, race/ethnicity, marital status, PIR, education level, energy intake, smoke, alcohol use, physical activity, BMI, hypertension, DM, hyperlipidemia, and CKD.

**Supplementary Table 4. Training set metrics for five machine learning models identifying prevalent CVD in patients with kidney stone disease.**

| **Model** | **AUC** | **Sensitivity** | **Specificity** | **Accuracy** | **Recall** | **Brier Score** |
| --- | --- | --- | --- | --- | --- | --- |
| LR | 0.821 | 0.753 | 0.751 | 0.752 | 0.753 | 0.166 |
| NB | 0.741 | 0.770 | 0.610 | 0.649 | 0.770 | 0.248 |
| RPART | 0.781 | 0.770 | 0.709 | 0.723 | 0.770 | 0.183 |
| KNN | 0.889 | 0.903 | 0.669 | 0.739 | 0.903 | 0.170 |
| RF | 0.867 | 0.812 | 0.769 | 0.780 | 0.812 | 0.159 |

Abbreviations: AUC, area under the curve; KNN, k-nearest neighbors; LR, logistic regression; NB, naive bayes; RF, random forest; RPART, recursive partitioning and regression tree

**Supplementary Table 5. Performance of logistic regression models for identifying prevalent CVD in patients with kidney stone disease using over-sampling and under-sampling preprocessing.**

|  | **AUC** | **Sensitivity** | **Specificity** | **Accuracy** | **Recall** | **Brier Score** | **F1 score** | **balanced accuracy** |
| --- | --- | --- | --- | --- | --- | --- | --- | --- |
| **Over-sampling** | 0.766 | 0.721 | 0.679 | 0.689 | 0.721 | 0.200 | 0.528 | 0.700 |
| **Under-sampling** | 0.784 | 0.705 | 0.707 | 0.706 | 0.705 | 0.192 | 0.536 | 0.706 |

Abbreviations: AUC, area under the curve.

**Supplementary Table 6. Performance metrics of the logistic regression model for identifying prevalent CVD across different demographic groups.**

| **Demographic groups** | **AUC** | **Sensitivity** | **Specificity** | **Accuracy** | **Recall** | **Brier Score** | **F1 score** | **balanced accuracy** |
| --- | --- | --- | --- | --- | --- | --- | --- | --- |
| Sex |  |  |  |  |  |  |  |  |
| Female | 0.718 | 0.585 | 0.842 | 0.796 | 0.585 | 0.169 | 0.511 | 0.714 |
| Male | 0.802 | 0.727 | 0.704 | 0.711 | 0.727 | 0.188 | 0.587 | 0.716 |
| Race |  |  |  |  |  |  |  |  |
| Mexican American | 0.826 | 0.563 | 0.935 | 0.839 | 0.563 | 0.132 | 0.643 | 0.749 |
| Non-Hispanic Black | 0.835 | 0.611 | 0.875 | 0.817 | 0.611 | 0.139 | 0.595 | 0.743 |
| Non-Hispanic White | 0.803 | 0.766 | 0.680 | 0.702 | 0.766 | 0.199 | 0.570 | 0.723 |
| Other Hispanic | 0.732 | 0.625 | 0.810 | 0.780 | 0.625 | 0.175 | 0.476 | 0.717 |
| Education level |  |  |  |  |  |  |  |  |
| Less than high school | 0.728 | 0.787 | 0.607 | 0.672 | 0.787 | 0.238 | 0.632 | 0.697 |
| High school or equivalent | 0.790 | 0.607 | 0.777 | 0.740 | 0.607 | 0.175 | 0.500 | 0.692 |
| Above high school | 0.792 | 0.648 | 0.818 | 0.784 | 0.648 | 0.157 | 0.543 | 0.733 |
| Marital status |  |  |  |  |  |  |  |  |
| Married | 0.803 | 0.662 | 0.789 | 0.759 | 0.662 | 0.165 | 0.563 | 0.726 |
| Never married | 0.750 | 0.667 | 0.818 | 0.786 | 0.667 | 0.157 | 0.571 | 0.742 |
| Living with partner | 0.713 | 0.500 | 0.900 | 0.786 | 0.500 | 0.180 | 0.571 | 0.700 |
| Other | 0.768 | 0.805 | 0.667 | 0.701 | 0.805 | 0.219 | 0.573 | 0.736 |

Abbreviations: AUC, area under the curve.

**Supplementary Table 7. Performance metrics of the logistic regression model for identifying prevalent CVD in the general population.**

| **Model** | **AUC** | **Sensitivity** | **Specificity** | **Accuracy** | **Recall** | **Brier Score** | **F1 score** | **Balanced accuracy** |
| --- | --- | --- | --- | --- | --- | --- | --- | --- |
| **LR** | 0.765 | 0.678 | 0.722 | 0.716 | 0.678 | 0.192 | 0.433 | 0.700 |

Abbreviations: AUC, area under the curve; LR, logistic regression.

**Supplementary Table 8. Performance metrics of the Q-Lite model for identifying prevalent CVD in patients with kidney stone disease.**

| **Model** | **AUC** | **Sensitivity** | **Specificity** | **Accuracy** | **Recall** | **Brier Score** | **F1 score** | **Balanced accuracy** | **P value (DeLong Test)^a^** |
| --- | --- | --- | --- | --- | --- | --- | --- | --- | --- |
| **Q-Lite** | 0.781 | 0.692 | 0.731 | 0.724 | 0.692 | 0.184 | 0.480 | 0.711 | 0.234 |

Abbreviations: AUC, area under the curve.

^a^. The P-value from the DeLong test comparing LR model and Q-Lite model.

**Supplementary Table 9. Performance comparison of weighted and unweighted logistic regression models for identifying prevalent CVD in patients with kidney stone disease.**

| **Model** | **AUC** | **Sensitivity** | **Specificity** | **Accuracy** | **Recall** | **Brier Score** | **F1 score** | **Balanced accuracy** |
| --- | --- | --- | --- | --- | --- | --- | --- | --- |
| **Unweighted LR model** | 0.801 | 0.721 | 0.771 | 0.759 | 0.721 | 0.169 | 0.590 | 0.746 |
| **Weighted LR model** | 0.758 | 0.604 | 0.844 | 0.791 | 0.604 | 0.148 | 0.543 | 0.724 |

Abbreviations: AUC, area under the curve; LR, logistic regression.

**Supplementary Table 10. Clinical significance and interpretation of predictive variables.**

| Variable | Clinical Significance | Underlying mechanisms |
| --- | --- | --- |
| Chest pain | Suggests existing or undiagnosed CVD | Classic symptom of myocardial ischemia or cardiovascular events |
| Age | Non-modifiable factor associated with CVD status | Aging leads to vascular and cardiac functional decline |
| Hypertension | \|  \| \| --- \|  \| Strong contributor to CVD status \| \| --- \| | Impairs endothelial function and accelerates atherosclerosis |
| BMI | Positively associated with CVD status | Reflects obesity and its association with multiple CVD risk factors |
| Sitting time | Positively associated with CVD status | Contributes to metabolic disorders and vascular dysfunction |
| Physical activity | Negatively associated with CVD status | Promotes cardiovascular health and reduces disease risk |
| PIR | Socioeconomic indicator associated with CVD status | Affects healthcare access and lifestyle choices |
| PUFA | Negatively associated with CVD status | Reduces inflammation and improves lipid metabolism |
| Folate | \|  \| \| --- \|  \| Negatively associated with CVD status \| \| --- \| | Lowers blood homocysteine levels and protects cardiovascular system |
| Magnesium | Negatively associated with CVD status | Reduces inflammation, enhances antioxidant activity, and improves endothelial function |
| Sex | Associated with differences in CVD status | Physiological and hormonal differences |
| Race | Associated with differences in CVD status | Genetic and environmental differences |
| Education level | \|  \| \| --- \|  \| Socioeconomic indicator associated with CVD status \| \| --- \| | Affects health knowledge and healthcare access |
| Heart disease family history | Associated with CVD status | Genetic susceptibility and shared lifestyle factors |

Abbreviations: BMI, body mass index; PIR, poverty income ratio; PUFA, polyunsaturated fatty acids.
